# MOP and NOP receptor interaction: Studies with a dual expression system and bivalent peptide ligands

**DOI:** 10.1371/journal.pone.0260880

**Published:** 2022-01-21

**Authors:** M. F. Bird, J. McDonald, B. Horley, J. P. O’Doherty, B. Fraser, C. L. Gibson, R. Guerrini, G. Caló, D. G. Lambert

**Affiliations:** 1 Department of Cardiovascular Sciences, Anaesthesia, Critical Care and Pain Management, University of Leicester, Leicester, United Kingdom; 2 Department of Neuroscience, Psychology and Behaviour, University of Leicester, Leicester, United Kingdom; 3 School of Psychology, University of Nottingham, Psychology Building, University Park, Nottingham, United Kingdom; 4 Department of Chemical, Pharmaceutical and Agricultural Sciences, University of Ferrara, Ferrara, Italy; 5 Department of Pharmaceutical and Pharmacological Sciences, University of Padova, Padova, Italy; University of Arizona College of Medicine, UNITED STATES

## Abstract

Opioids targeting mu;μ (MOP) receptors produce analgesia in the peri-operative period and palliative care. They also produce side effects including respiratory depression, tolerance/dependence and addiction. The N/OFQ opioid receptor (NOP) also produces analgesia but is devoid of the major MOP side effects. Evidence exists for MOP-NOP interaction and mixed MOP-NOP ligands produce analgesia with reduced side effects. We have generated a HEK_MOP/NOP_ human expression system and used bivalent MOP-NOP and fluorescent ligands to (i) probe for receptor interaction and (ii) consequences of that interaction. We used HEK_MOP/NOP_ cells and two bivalent ligands; Dermorphin-N/OFQ (MOP agonist-NOP agonist; DeNO) and Dermorphin-UFP101 (MOP agonist-NOP antagonist; De101). We have determined receptor binding profiles, GTPγ[^35^S] binding, cAMP formation and ERK1/2 activation. We have also probed MOP and NOP receptor interactions in HEK cells and hippocampal neurones using the novel MOP fluorescent ligand, Dermorphin_ATTO488_ and the NOP fluorescent ligand N/OFQ_ATTO594_. In HEK_MOP/NOP_ MOP ligands displaced NOP binding and NOP ligands displaced MOP binding. Using fluorescent probes in HEK_MOP/NOP_ cells we demonstrated MOP-NOP probe overlap and a FRET signal indicating co-localisation. MOP-NOP were also co-localised in hippocampal tissue. In GTPγ[^35^S] and cAMP assays NOP stimulation shifted the response to MOP rightwards. At ERK1/2 the response to bivalent ligands generally peaked later. We provide evidence for MOP-NOP interaction in recombinant and native tissue. NOP activation reduces responsiveness of MOP activation; this was shown with conventional and bivalent ligands.

## Introduction

Opioids targeting the mu opioid peptide (MOP) receptor are amongst the most widely used analgesics in both acute and chronic pain treatment. However, exclusively targeting this receptor leads to addiction and/or tolerance. More interestingly, animal studies demonstrated that co-targeting an additional member of the opioid receptor family with either agonists or antagonists has been shown to be beneficial in longer-term antinociceptive paradigms, while reducing tolerance [1]. This synergistic effect is believed to be derived from the ability of opioid receptors to interact at a molecular and structural level [2]. The dimerisation of opioid receptors has been shown to influence receptor trafficking, signalling and the ability to inhibit or delay tolerance and dependence/addiction [3–5]. With regards to heterodimeric pairings, the majority of work in this field has been based on the association between MOP and delta opioid peptide (DOP) receptors [6–13]. There is growing evidence to suggest that the potential pairing of the MOP and nociceptin/orphanin FQ (N/OFQ) peptide (NOP) receptor systems may be beneficial in developing analgesics with reduced side effects [14–17].

NOP is a “non-classical” opioid receptor lacking affinity for the opioid antagonist naloxone [18] and with little or no affinity for the classical endogenous opioid peptides. The NOP receptors endogenous ligand N/OFQ has no affinity for classical opioid receptors [19]. Initial studies in rodents indicated that the NOP-N/OFQ system was pronociceptive when administered supraspinally and antinociceptive when administered spinally, however recent work in non-human primates (NHP) has shown these effects to be species dependent, with N/OFQ providing potent analgesia at both sites [20–23].

Evidence linking MOP-NOP is supported from *in vitro* experiments to *in vivo* work with NHP demonstrating the synergistic effect N/OFQ has on morphine administration [24]. The most recent work spanning both *in vivo* and *in vitro* studies centres on the development of a mixed MOP-NOP ligand, cebranopadol. Cebranopadol has a favourable safety profile in pre-clinical studies [17]. This complements data with MOP-NOP ligands BU08028 [25] and AT-121 [26]. Cebranopadol is being evaluated in clinical trials [27–29].

The co-operative effect seen in the dual targeting of MOP and NOP could be due to a number of factors, including the formation of MOP/NOP heterodimers, with this action being supported by the co-localisation of MOP and NOP throughout the pain pathway [30–32]. While work remains to demonstrate evidence of MOP-NOP heterodimerisation *in vivo*, *in* vitro studies suggest heterodimerisation. An elegant study by Evans et.al., using tagged receptors, demonstrated the co-localisation of NOP and MOP (as well as DOP and KOP receptors) [33]. Treatment of these co-expressing cell lines with N/OFQ demonstrated co-internalisation of both the classical opioid receptor and the NOP receptor indicating their structural linkage [33]. Work in CHO cells demonstrated co-immunoprecipitation of NOP and MOP opioid receptors, as well as the ability of classical opioid ligands to displace [^3^H]-N/OFQ, which does not occur in single expression systems [34]. Heterodimerisation of MOP and NOP has also been shown to negatively impact MOP cell signalling [35] but can lead to an improved side effect profile, as noted by heterodimerisation of the truncated 6TM MOP receptor to NOP [36].

Previous work from our group has identified a novel bivalent ligand, Dermorphin-Nociceptin/Orphanin FQ (DeNO) [15] that functions as an agonist at both MOP and NOP. To further understand the role NOP may play in MOP receptor signalling, we have synthesised a novel MOP agonist (Dermorphin)-NOP antagonist (UFP-101) bivalent ligand (De101). As with DeNO, the MOP agonist chosen was Dermorphin. UFP-101 ([Nphe^1^,Arg^14^,Lys^15^]N/OFQ-NH_2_), is an antagonist analogue of N/OFQ designed to display improved affinity and duration of action *in vivo* [37]. UFP-101 displays high affinity for the NOP receptor (pK_i_ of 10.14) with an antagonist pA_2_ value in the range 8.4–9 [38].

While the effects of selective peptide agonists, such as DAMGO and N/OFQ [5,10,33,35], have been studied in co-expression systems, very little evidence exists demonstrating the activity of mixed ligands in a co-expression system. In order to assess the cellular aspects of mixed ligand interactions in a system co-expressing MOP and NOP, we have developed a human HEK co-expression system (HEK_MOP/NOP_). We have used the bivalent MOP/NOP full agonist DeNo (15), in conjunction with the newly synthesised De101. De101 is a counterpoint to the ligand DeNO (**S4 Fig in S1 File**). Binding and functional activity assays were performed to determine the suitability of De101 as a test ligand. Furthermore, we visualised and determined co-expression using two novel fluorescent ligands, N/OFQ_ATTO594_ [14] for NOP and Dermorphin_ATTO488_ for MOP [39]. These imaging experiments were performed both using HEK_MOP/NOP_ and mouse CA1 hippocampal neurons to determine the potential for receptor co-localisation *Ex Vivo*. Mouse CA1 hippocampal neurones have been shown to express both MOP [40] and NOP receptors [41], however colocalisation/coexpression has not yet been definitively proven.

We hypothesise that co-expression of MOP and NOP will lead to measurable changes in downstream signalling pathways, such as GTPγ[^35^S] binding and cAMP inhibition, as well as changes in the activation of the mitogen-activated protein kinase, ERK1/2.

## Materials and methods

### Materials

Dermorphin, N/OFQ, UFP-101, DeNO, De101, N/OFQ_ATTO594_ and Dermorphin_ATTO488_ were synthesised in house (University of Ferrara). Tritiated N/OFQ ([^3^H]-N/OFQ), tritiated diprenorphine ([^3^H]-DPN), GTPγ[^35^S] and [^3^H]-cyclic adenosine monophosphate ([^3^H]-cAMP) were purchased from Perkin Elmer (UK). Naloxone-HCL, cyclic adenosine monophosphate (cAMP), GTPγS were purchased from Sigma-Aldrich (U.K). Phospho-ERK1/2 [42], vinculin [43] and Anti-NeuN [44] primary antibodies were purchased from Cell Signalling Technologies (U.S.A). All cell culture products were purchased from Thermofisher Scientific (UK). Ab-Delivirin™ purchased from OZ Bioscience (Australia), anti-rabbit secondary conjugated to Alexa-405 [45] was purchased from Abcam (U.K). All other reagents were of the highest purity available. CHO_MOP/DOP/KOP/NOP_ cells were a gift from T Costa (Instituto Superiore di Sanita, Rome, Italy).

### Cell culture

Chinese hamster ovary (CHO) cells expressing recombinant human opioid receptors were cultured in either Hams F12 (for classical opioid receptors) or DMEM/Hams F12 1:1 (for CHO_NOP_ cells). The media contained 10% fetal bovine serum, 100 IU ml−1 penicillin, 100 μg ml−1 streptomycin and 2.5 μg ml−1 fungizone. Stock cultures were maintained through the addition of 200 μg ml−1 G418 (for CHO_MOP_, CHO_DOP_ and CHO_KOP_ cells). CHO_NOP_ cells were maintained with G418 (200 μg ml−1) and hygromycin B (200 μg ml−1). Human embryonic kidney (HEK) cells expressing the recombinant human opioid receptors were grown in MEM containing 100 μg.ml^-1^ streptomycin, 2.5 μg.ml^-1^ fungizone, 100 IU.ml^-1^ penicillin and 10% foetal bovine serum. G418 (400 μg.ml^-1^) was used to maintain cells expressing human MOP receptors, with hygromycin B (400 μg.ml^-1^) used for cells expressing recombinant NOP receptors. For the cells co-expressing MOP and NOP, a combination of G418 (400 μg.ml^-1^) and hygromycin B (200 μg.ml^-1^) was used. A temperature of 37°C was used to maintain cell cultures in 5% CO_2_/humidified air. Cells were used for experiments upon reaching confluency as in [15].

### Development of Co-expression system

Plasmids containing cDNA clones for human MOP or human NOP receptors were purchased from cDNA Resource Centre (www.cdna.org). Clones were individually transfected into HEK293 cells using Fugene HD (Promega, UK). Cell lines stably expressing the MOP receptor or NOP receptor were selected by adding geneticin (800μg/ml for MOP) or hygromycin B(600μg/ml for NOP) to cell culture medium. Following selection of preferred HEK_MOP_ single expression clone, cells were co-transfected with NOP, with co-expression selected by co-administering geneticin (400μg.ml^-1^) and hygromycin B (200μg.ml^-1^). Surface expression of receptors for the single and co-expression systems was determined by using radioligand binding assays as described below (further cloning details can be found in **Supplement section 1 in S1 File**).

### Synthesis of De101

The MOP/NOP ligand De101 was synthesized using a classical thiol-Michael reaction following the experimental conditions previously reported for the synthesis of DeNo using [Cys^18^]UFP-101 in place of [Cys^18^]N/OFQ-NH_2_. Structure and Chemistry can be found in **S4-S6 Figs in S1 File.**

### Membrane preparation

CHO_OPIOID_ or HEK_OPIOID_ cells were harvested, homogenised and resulting membrane fragments resuspended in wash buffer (50mM Tris-HCl and 5mM MgSO_4_, pH 7.4 with KOH) for saturation and displacement assays or a homogenisation buffer (50mM HEPES and 1mM EDTA, pH 7.4 with NaOH) for GTPγ[^35^S] functional assays. Membrane suspensions were sedimented at 20,374g for 10 minutes at 4°C, with the process being repeated three times. The pellet was suspended in the appropriate buffer at the desired volume before protein concentration was measured using a Lowry assay [15,46].

### Radioligand binding assays

In Saturation binding assays, a range of concentrations of [^3^H]-DPN (HEK_MOP_ or HEK_MOP/NOP_) or [^3^H]-N/OFQ (HEK_NOP_ or HEK_MOP/NOP_) were incubated with membrane protein (40 μg) in a Tris-HCL based buffer (0.5ml of 50 mM Tris, 0.5% BSA, pH 7.4). Non-specific binding was determined by using 10μM naloxone and/or 1μM N/OFQ.

For displacement binding assays, membrane protein (40 μg) was incubated in 0.5ml of 50 mM Tris, 0.5% BSA and ~0.8nM [^3^H]-DPN (CHO_hMOP/DOP/KOP_, HEK_MOP_ or HEK_MOP/NOP_) or ~0.8nM [^3^H]-N/OFQ (HEK_NOP_ or HEK_MOP/NOP_) along with varying concentrations (10 μM-1pM) of the test ligands. Non-specific binding was determined using 10μM naloxone and/or 1μM N/OFQ.

In both saturation binding and displacement binding studies, samples were incubated for 1 hr at room temperature before reactions were terminated by vacuum filtration onto polyethylenimine (PEI) soaked Whatman-GF/B filters, using a Brandel Harvester [14].

### GTPγ[^35^S] assays

In GTPγ[^35^S] functional assays, 25μg of membrane protein was incubated in 50mM HEPES, 1mM EDTA, 1mM DTT, 5mM MgCl_2_, 100mM NaCl 0.1% BSA, 0.15 mM bacitracin; pH 7.4, GDP (33 μM), and ∼150 pM GTPγ[^35^S]. Varying concentrations (10 μM-1pM) of the test ligands were added prior to incubation. Unlabelled GTPγS (10μM) was used to determine non-specific binding. The samples were incubated for 1hr at 30°C with gentle agitation, following which, reactions were terminated by vacuum filtration through Whatman-GF/B filters (without PEI), using a Brandel harvester [15].

### Cyclic adenosine monophosphate inhibition assays

HEK_MOP_, HEK_NOP_ or HEK_MOP/NOP_ cells were suspended in Krebs/HEPES buffer, containing isobutylmethylxanthine (1mM) and forskolin (1μM) as appropriate. Varying concentrations (0.1pM-1μM) of the test ligands were added prior to incubation. Following a 15 minute incubation at 37°C, the reaction was terminated by addition of 10M HCl, following which 10M NaOH and Tris-HCl (1M, pH7.4) were added to neutralise the reaction followed by centrifugation (13,000g, 2 min). Binding protein from bovine adrenal cortex was used to measure cAMP collected from the supernatant as in [47].

### ERK 1/2 phosphorylation assays

Western blotting was used to determine ERK1/2 activity in HEK_MOP_, HEK_NOP_ or HEK_MOP/NOP_ cells. Cells were serum starved for 24 hours prior to treatment with the test ligands. Media was removed after 24 hours and replaced with Krebs/HEPES buffer. All test ligands were delivered at a concentration of 1μM. Ligands were added at appropriate time points (2.5; 5; 7.5; 10; 15; 20 and 30 minute intervals), following which the assay was terminated by removal of Krebs/HEPES and addition of lysis buffer (Tris-HCl (pH 7.4), 20 mM; 1% (vol/vol); Triton X-100, 10% (vol/vol); glycerol, NaCl, 137 mM; EDTA, 2 mM; β-glycerophosphate, 25 mM; sodium orthovanidate; 1 mM; phenylmethanesulfonylfluoride, 500μM; leupeptin, 0.1 mg/ml; benzamidine, 0.2 mg/ml; pepstatin, 0.1 mg/ml). Cell wells were scraped, the lysis buffer collected and centrifuged at 17,000g at 0°C for 10 minutes.

The supernatant was removed and added to an equal volume of Laemmli buffer (100mM Tris-HCl (pH 6.8), 2% SDS, 10% Glycerol, 0.1% Bromophenol Blue). Samples were denatured (100°C for 5 minutes) and separated by 10% SDS-PAGE, followed by transfer onto nitrocellulose paper by wet transfer (39mM glycine, 48mM Tris-Base, 0.037%w/v SDS, 20% methanol). Membranes were blocked in 5% milk TBS-T ([50 mm Tris-base, 150 mm NaCl, 0.1% Tween-20 (vol/vol), pH 7.5 NaOH) solution for 1 hr at room temperature under gentle agitation. Membranes were washed 3 times in TBS-T, following which the membranes were incubated in primary phospho-ERK1/2 antibodies (1:6000 dilution) diluted in TBS-T overnight at 4°C. Following 3 washes in TBS-T, membranes were incubated in horseradish peroxidase-conjugated secondary antibodies (1 hr room temperature; 1:1000 dilution 5% milk/TBS-T solution). Chemiluminescence detection, using the ChemiDoc™ MP imaging system (Bio-Rad, UK) was used to visualise immune-reactive bands.

In order to probe for loading controls, membranes were stripped using Restore Plus™ (ThermoFisher, UK) for 15 minutes. Membranes were thoroughly washed in TBS-T and blocked using the 5% milk/TBS-T for 1 hr at room temperature. Membranes were again probed overnight at 4°C, using ERK1/2 (total) antibody (1:3000 dilution in TBS-T) following which detection of the immune-reactive band was achieved as previously described. Normalisation of total protein levels was achieved by representing levels of phosphor-ERK1/2 as a proportion of total ERK1/2 protein [15].

### Mouse hippocampal brain slices

This study was conducted in accordance with the UK Animals Scientific Procedures Act, 1986 and following approval by the animal welfare and ethics committee of the University of Leicester. Pregnant C57/BL6 mice, at stage between E11-E13, were obtained from Charles River UK and delivered in house. Hippocampal slices were prepared from male and female C57/BL6 mouse pups aged between postnatal day (P) 6 to 9. Mice were housed in a 12 hour light/dark cycle and food and water was given ad libitum. Mice were humanely killed in accordance with home office guidelines by schedule 1 using cervical dislocation followed by swift decapitation.

Hippocampal slices were prepared by the methods denoted in [48], with further modifications to promote neural outgrowths. Dissection buffer (DB) utilised for dissections consisted of HBSS (Hank balanced salt solution), 4.5mg/ml glucose and 3.75μg/ml of amphotericin B. Culture media contained 50% MEM, (minimal essential medium), 25% HBSS, 25% heat inactivated horse serum, 4.5mg/ml glucose, 3.75μg/ml of amphotericin B and 0.5 mM glutamine. Following dissection, brains were transferred to a sterile petri dish on ice containing ice cold DB. After separation of the hemispheres with a scalpel, the hippocampi were isolated whilst the tissue was fully submerged in ice cold DB. Transverse hippocampal slices at 350μm were prepared using a tissue chopper (McIlwain tissue chopper). Using sterile syringe needles, slices were carefully separated and transferred into 6 well plates containing 28mm Menzel glaser #1-coverslips (Thermos Scientific, UK) with the prior addition of Celltak™ (1μg.mL1) (Sigma, UK). This promotes adhesion of slices and neuronal outgrowth. Slices were submerged in 1ml of pre-warmed media. Plates were placed in a humidified incubator at 37°C and perfused with 95% O_2_ and 5%CO_2_ for a minimum of one week prior to inspection for neuronal outgrowths. Culture media was topped up every 2–3 days. Twenty-four hours prior to experimental use, cells were treated with a complex containing anti-Neun Ab attached to an anti-rabbit Alexa-fluor 405nm secondary Ab (UK) coated in 10μL Ab-Delivirin™ to allow identification and visualisation of neuronal cells [41].

### Confocal microscopy

Confluent HEK_MOP_, HEK_NOP_ and HEK_MOP/NOP_ cells (and hippocampal slices) were grown on ethanol-sterilised coverslips (28mm Menzel glaser #1), incubated for 24h before being transferred to a Harvard PDMI-2 peltier unit and continually perfused with ice-cold Krebs buffer, pH 7.4, maintaining a constant temperature of 4°C.

N/OFQ_ATTO594_ and Derm_ATTO488_ were injected either separately or together at a concentration of 100nM following which images were acquired using a Nikon C1Si confocal microscope (60X oil immersion objective). N/OFQ_ATTO594_ (excitation 594 nm; emission 620 nm), and/or Derm_ATTO488_ (excitation 488 nm; emission 520 nm) were allowed to incubate for 5 min, before coverslips were washed with the ice-cold Krebs. Following wash-off, cells were imaged at the desired wavelength (Derm_ATTO488_-488nm, image capture at 520 nm; N/OFQ_ATTO594_- 594 nm, image captured at 620nm), with the images collected by Nikon C1Si software [14,39]. As both probes are agonists all experiments were undertaken at 4°C to prevent receptor activation and internalisation.

FRET studies were undertaken by measuring the binding of both 100nM Derm_ATTO488_ and 100nM N/OFQ_ATTO594_ on HEK_MOP/NOP_ cells. Derm_ATTO488_ was stimulated by the 488nm laser (laser power: 20%; Gain: 6.85) with measurements assessed in both the green and red channel [49]. To confirm FRET-pairing, several controls were undertaken (**see Fig 7**). N/OFQ_ATTO594_ was incubated on HEK_MOP/NOP_ cells alone and measured with the 488 nm laser, while photobleaching of N/OFQ_ATTO594_ was undertaken using 594nm laser (laser power: 50% Gain: 7.10) in the presence of Derm_ATTO594_ to measure its relative fluorescence pre and post photobleaching [50]. All FRET experiments were undertaken at 4°C to prevent receptor activation and internalisation.

### Statistical analysis

For radioligand assays and Western blot techniques all data are expressed as the mean ± SEM (n). GraphPad Prism V6.05 (San Diego, USA) was used for curve fitting and analysis. For displacement binding studies, the concentration which caused 50% displacement of the radioligand (IC_50_) was corrected for the competing mass of radioligand by use of the Cheng and Prusoff equation using the pK_d_ values produced from saturation binding experiments (**S2, S4 and S6 Tables in S1 File**) [51]. GTPγ[^35^S] binding results are expressed as a stimulation factor (agonist stimulated specific binding/basal specific binding) [15]. In cAMP inhibition studies, results are expressed as percentage inhibition of forskolin stimulation. For ERK1/2 activity, following normalisation of the protein bands, ERK1/2 activity is measured as activity compared to basal levels. Statistical analysis was performed using t-test or one way ANOVA with Bonferroni correction, as described in figure and table legends. For Confocal microscopy, FIJI was used to analyse colocalization using the Ezcolocalization plugin to determine Pearson correlation coefficient [52]. This software measures relative overlapping fluorescence intensities to produce statistical data (Pearson coefficient correlation) to determine levels of co-expression. Changes in fluorescence during photobleaching experiments was measured as previously described using corrected total cell fluorescence [Corrected total cell fluorescence = Integrated density − (Area of selected cell × Mean fluorescence of background readings)] [53].

## Results

### Characterisation of De101

**Fig 1:**
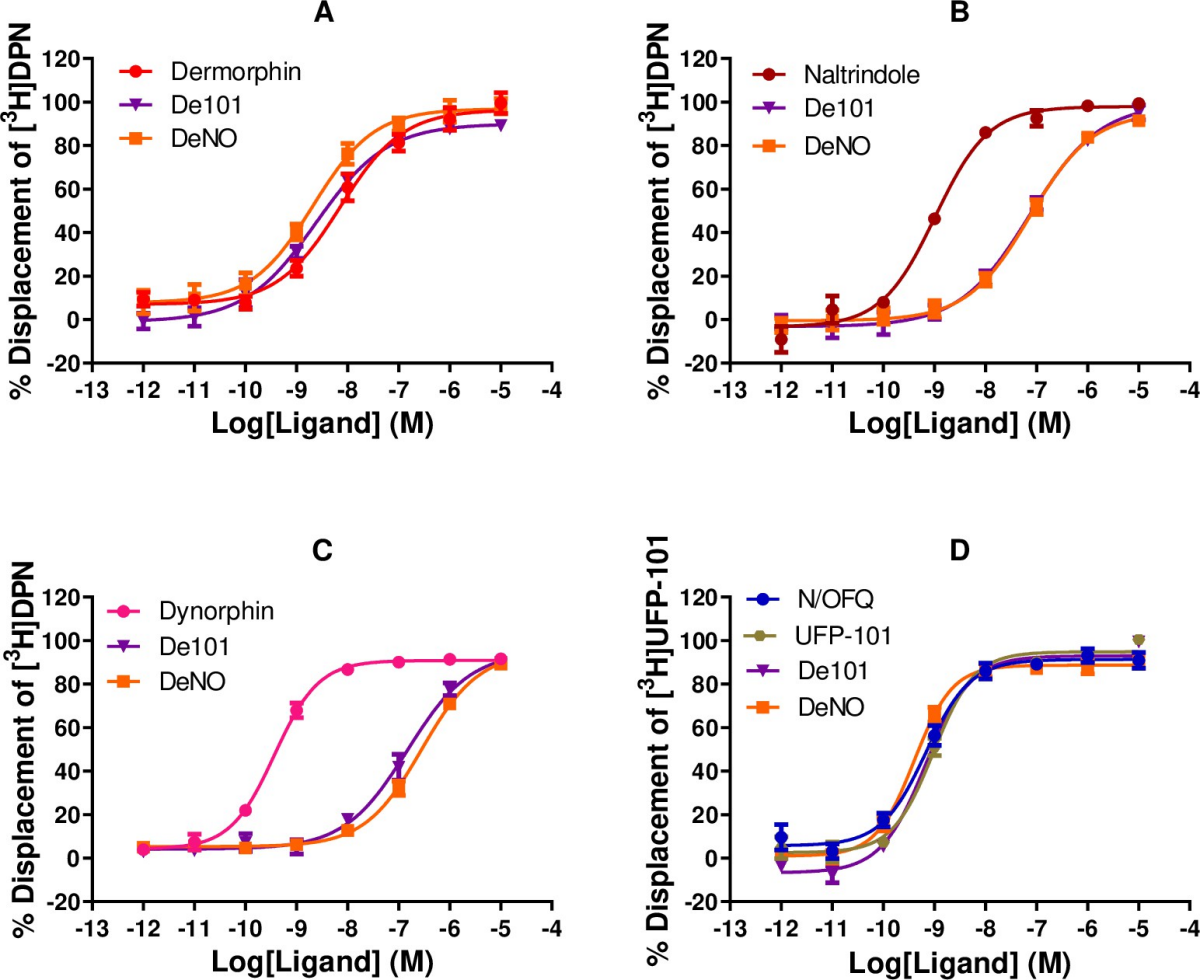
Displacement of [^3^H]-DPN by Dermorphin and De101 and reference ligands at (A) CHO_MOP_, (B) CHO_DOP_, (C) CHO_KOP_ and the displacement of [^3^H]UFP-101 by De101 and N/OFQ at (D) CHO_NOP_ cell membranes. Data are the mean (±SEM) of n = 5 experiments. DeNO curves are replicated from previous studies [14]. Reference ligands: Dermorphin; Naltrindole; Dynorphin-A; N/OFQ, (Nociceptin/Orphanin FQ).

In displacement binding studies at CHO_hMOP_, Dermorphin and De101 displaced the binding of [^3^H]-DPN in a concentration dependent and saturable manner (**Table 1 and Fig 1**). De101 (9.64) demonstrated a significant increase in affinity at MOP, when compared to the parent compound Dermorphin (8.69). At CHO_NOP_, De101 displaced [^3^H]UFP-101 in a concentration dependent and saturable manner. De101 (10.08) displayed similar pK_i_ values, for NOP, to the reference compounds N/OFQ (10.69) and UFP-101 (10.08).

**Table 1:** The pK_i_ values for both the reference ligands, Dermorphin, N/OFQ, UFP-101 and De101.

	CHO_MOP_	CHO_NOP_	CHO_DOP_	CHO_KOP_
Reference ligand	-	-	10.02 _(±0.26)_	10.16 _(±0.02)_
Dermorphin	8.69 _(±0.10)_	<5	7.17 _(±0.11)_	<5
N/OFQ	<5	10.69 _(±0.10)_	<5	<5
UFP-101	<5	9.87_(±0.10)_	<5	<5
DeNO	9.55 _(±0.10)_*	10.22 _(±0.09)_	8.12 _(±0.11)_*	7.34_(±0.13)_*
De101	9.64 _(±0.13)_*	10.08 _(±0.08)_	7.95 _(±0.13)_*	7.61_(±0.06)_*

Data are displayed as mean (±SEM) of n≥5 experiments. Statistical significance (*) demonstrates p<0.05, using one-way ANOVA with Bonferroni corrections, when compared to the reference ligands: Dermorphin (MOP), N/OFQ (NOP), Naltrindole (DOP) and Dynorphin-A (KOP).

At CHO_DOP_, De101 (7.95) demonstrated an increase in affinity compared to its parent compounds. Dermorphin displayed an affinity of 7.17, while UFP-101 failed to displace [^3^H]-DPN at the DOP receptor. Furthermore, De101 (7.61) showed affinity for the KOP receptor, whereas the parent compounds (Dermorphin and UFP-101) failed to displace [^3^H]-DPN at this receptor.

**Fig 2:**
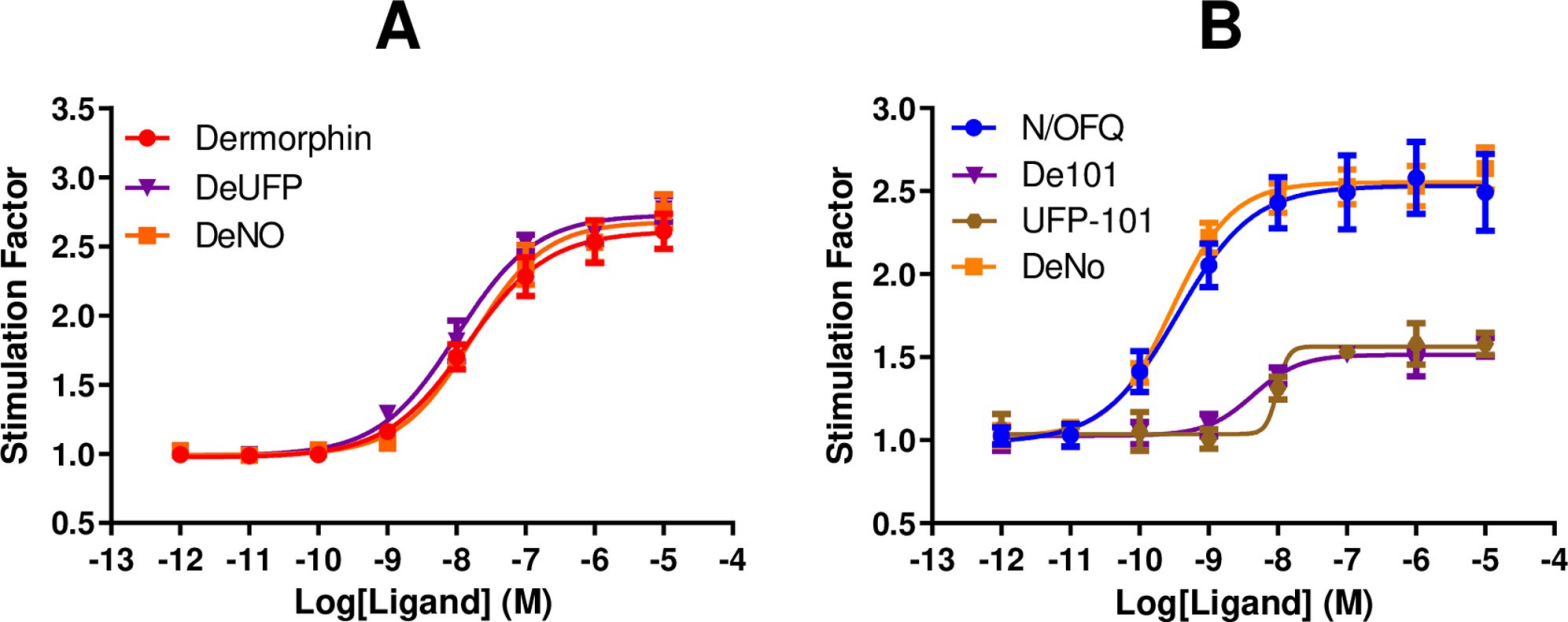
A) Ligand stimulated GTPγ[^35^S] binding by Dermorphin and De101 are shown in CHO_MOP_ cell membranes. B) Ligand stimulated GTPγ[^35^S] binding by N/OFQ, UFP-101 and De101 are shown in CHO_NOP_ cell membranes. DeNO curves are replicated from previous studies [14]. Data are the mean (±SEM) of n = 5 experiments.

Dermorphin and De101 stimulated the binding of GTPγ[^35^S] in a concentration dependent and saturable manner at the MOP receptor (**Fig 2A**). De101 (2.71) demonstrated similar maximal responses to that of Dermorphin (2.63). The pEC_50_ value for De101 (8.02) showed no significant difference to that of the parent compound, Dermorphin (7.83).

At CHO_NOP_, N/OFQ, UFP-101 and De101 stimulated the binding of GTPγ[^35^S] in a concentration dependent and saturable manner (**Fig 2B**). De101 (1.31) demonstrated an E_max_ similar to that of UFP-101 (1.42). Furthermore, De101 (8.11) demonstrated a similar pEC_50_ to that of its parent compound, UFP-101 (8.23).

### Characterisation of Ligands in the co-expression system

#### Saturation binding assays

**Fig 3:**
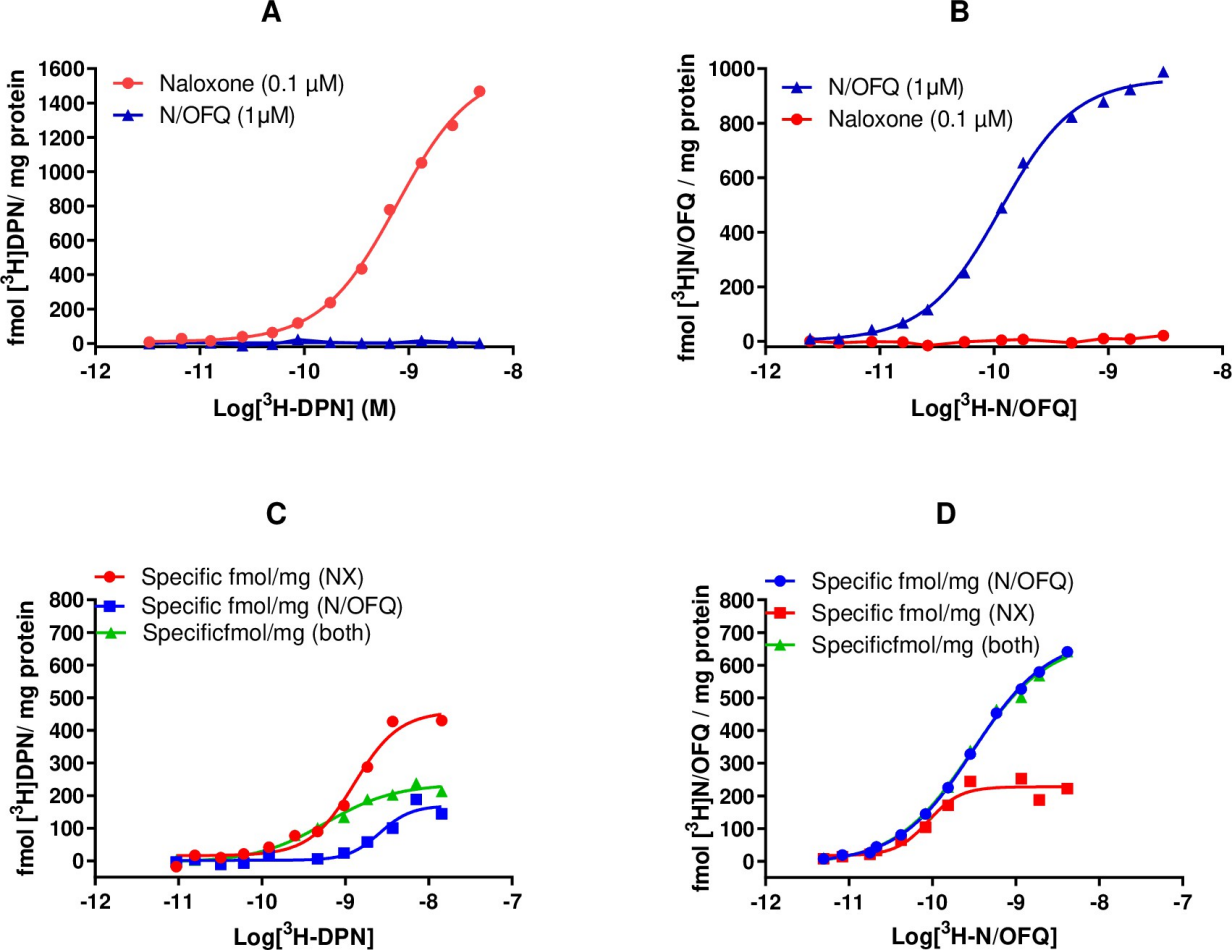
Representative saturation binding curves of A) [^3^H]-DPN binding in HEK_MOP_ cell membranes B) [^3^H]-N/OFQ binding in HEK_NOP_ cell membranes. Representative saturation binding curves C) [^3^H]-DPN binding or D) [^3^H]-N/OFQ binding in HEK_MOP/NOP_ cell membranes. Data are the mean (±SEM) of n = 5 experiments.

In saturation binding studies, both HEK_MOP_ (B_max_: 1542±35; pK_d_: 9.26±0.05 using [^3^H]-DPN) and HEK_NOP_ (B_max_: 887±93; pK_d_: 9.50±0.09 using [^3^H]-N/OFQ) demonstrated binding for their respective radioligands (with non-specific binding determined by naloxone for MOP or N/OFQ for NOP) (**Fig 3**). Neither Tritiated-DPN nor [^3^H]-N/OFQ were able to bind to HEK_NOP_ or HEK_MOP_ respectively **(Fig 3A and 3B)**.

In HEK_MOP/NOP_ cells, both [^3^H]-DPN and [^3^H]-N/OFQ were able to bind and both were displaced by naloxone, N/OFQ or a combination of both **(Fig 3C and 3D)**. Tritiated-DPN, in combination with naloxone, produced a B_max_ of 464 and pK_d_ of 8.72. Tritiated-DPN, with N/OFQ as the NSB, produced a B_max_ of 169 and pK_d_ of 8.59. When using [^3^H]-N/OFQ and N/OFQ to determine non-specific binding, a B_max_ of 689 and pK_d_ of 9.51 was produced. Tritiated-N/OFQ, with naloxone as NSB, produced a B_max_ of 228 and pK_d_ of 10.05_._ Cross displacement, in terms of NSB determination, suggests an interaction; this was probed further in a series of displacement experiments.

#### Displacement binding assays

**Fig 4:**
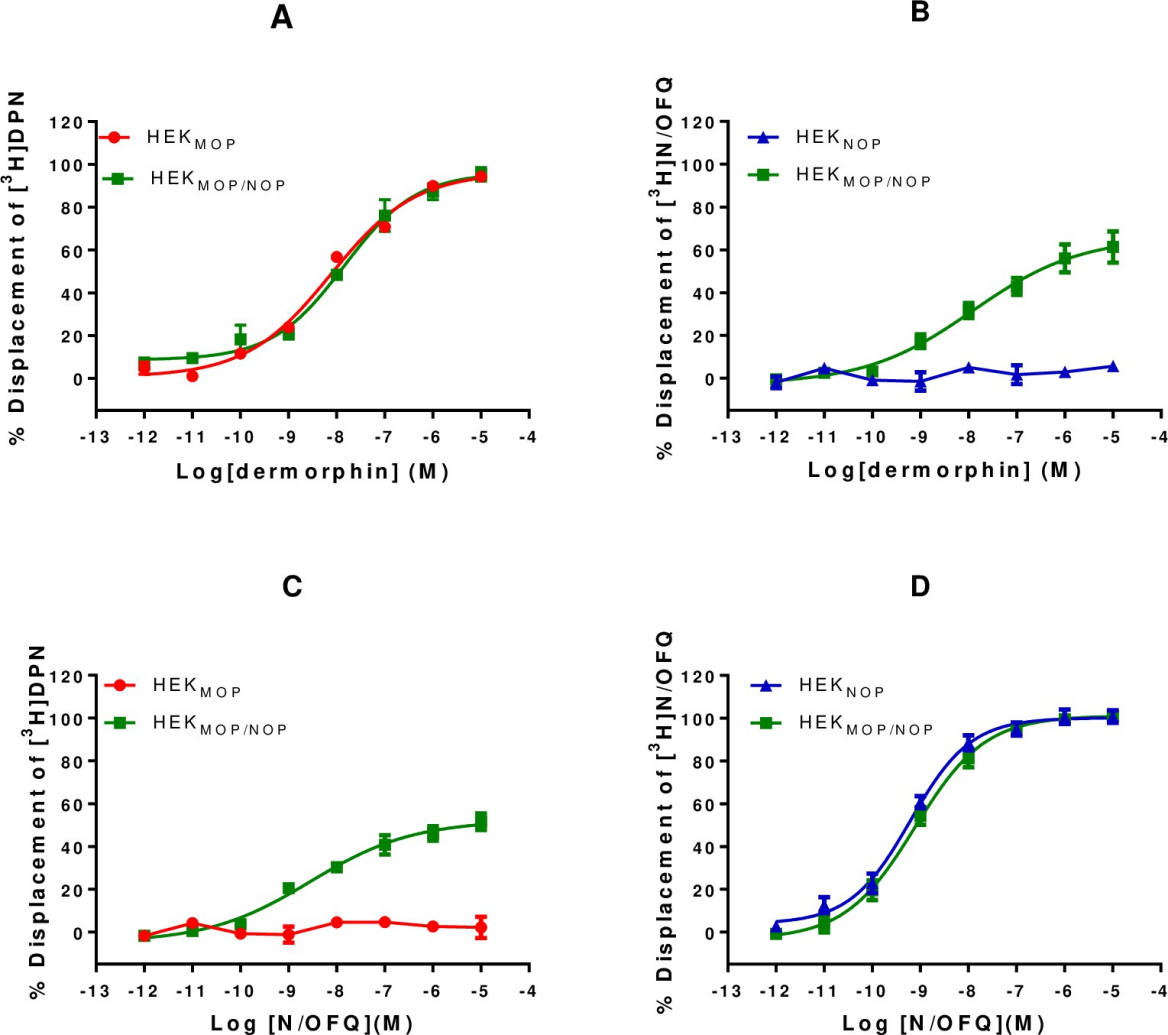
Displacement binding curves of A) Dermorphin in HEK_MOP_ and HEK_MOP/NOP_ cell membranes against [^3^H]-DPN or B) in HEK_NOP_ and HEK_MOP/NOP_ cell membranes against [^3^H]-N/OFQ. Displacement binding curves of C) N/OFQ in HEK_MOP_ and HEK_MOP/NOP_ cell membranes against [^3^H]-DPN or D) in HEK_NOP_ and HEK_MOP/NOP_ cell membranes against [^3^H]-N/OFQ. Data are the mean (±SEM) of n = 5 experiments.

Dermorphin displaced [^3^H]-DPN in HEK_MOP_ cells (pK_i_: 8.32) and in HEK_MOP/NOP_ cells (pK_i_: 7.86), with a small (but not statistically significant) reduction in affinity **(Fig 4 and Table 2)**. Dermorphin was unable to displace [^3^H]-N/OFQ in HEK_NOP_ cells, but was able to displace this radioligand in the co-expression system (pK_i_: 7.91) **(Fig 4 and Table 2)** displaying similar affinity when displacing [^3^H]-DPN in this cell line.

**Table 2:** The pK_i_ values for Dermorphin, N/OFQ, DeNO and De101 in HEK_MOP_, HEK_NOP_ and HEK_MOP/NOP_.

	HEK_MOP_	HEK_NOP_	HEK_MOP/NOP_	HEK_MOP/NOP_
	[^3^H]-DPN	[^3^H]-N/OFQ	[^3^H]-DPN	[^3^H]-N/OFQ
Dermorphin	8.32 _(±0.15)_	No Binding	7.86 _(±0.14)_	7.91_(±0.22)_^d^
N/OFQ	No Binding	9.39 _(±0.07)_	8.75_(±0.03)_^c^	9.11 _(±0.09)_
DeNO	9.00 _(±0.11)_^a^	9.69 _(±0.11)_	8.44 _(±0.16)_^c^	9.59 _(±0.17)_^d^
De101	9.28 _(±0.11)_^a^	8.68 _(±0.08)_^b^	8.63 _(±0.03)_^c^	9.24 _(±0.04)_^d^

^a^significant difference vs Dermorphin (p≤0.05).

^b^significant difference to the reference ligand, N/OFQ, (p≤0.05).

^c^significant difference to the reference ligand, Dermorphin, in HEK_MOP/NOP_ (p≤0.05).

^d^significant difference to the reference ligand, N/OFQ, in HEK_MOP/NOP_ (p≤0.05). Data are displayed as mean (±SEM) of n = 5 experiments. p≤0.05 (ANOVA) followed by post hoc Bonferroni multiple comparisons.

N/OFQ displaced [^3^H]-N/OFQ in HEK_NOP_ cell membranes (pK_i_: 9.39) and in the HEK_MOP/NOP_ co-expression system with a pK_i_ of 9.11 **(Fig 4 and Table 2)**. N/OFQ was unable to displace [^3^H]-DPN in HEK_MOP_ cell membranes but did displace this radioligand in the co-expression system with a pK_i_ of 8.75; displaying similar affinity to that achieved in the co-expression system when using [^3^H]-N/OFQ.

**Fig 5:**
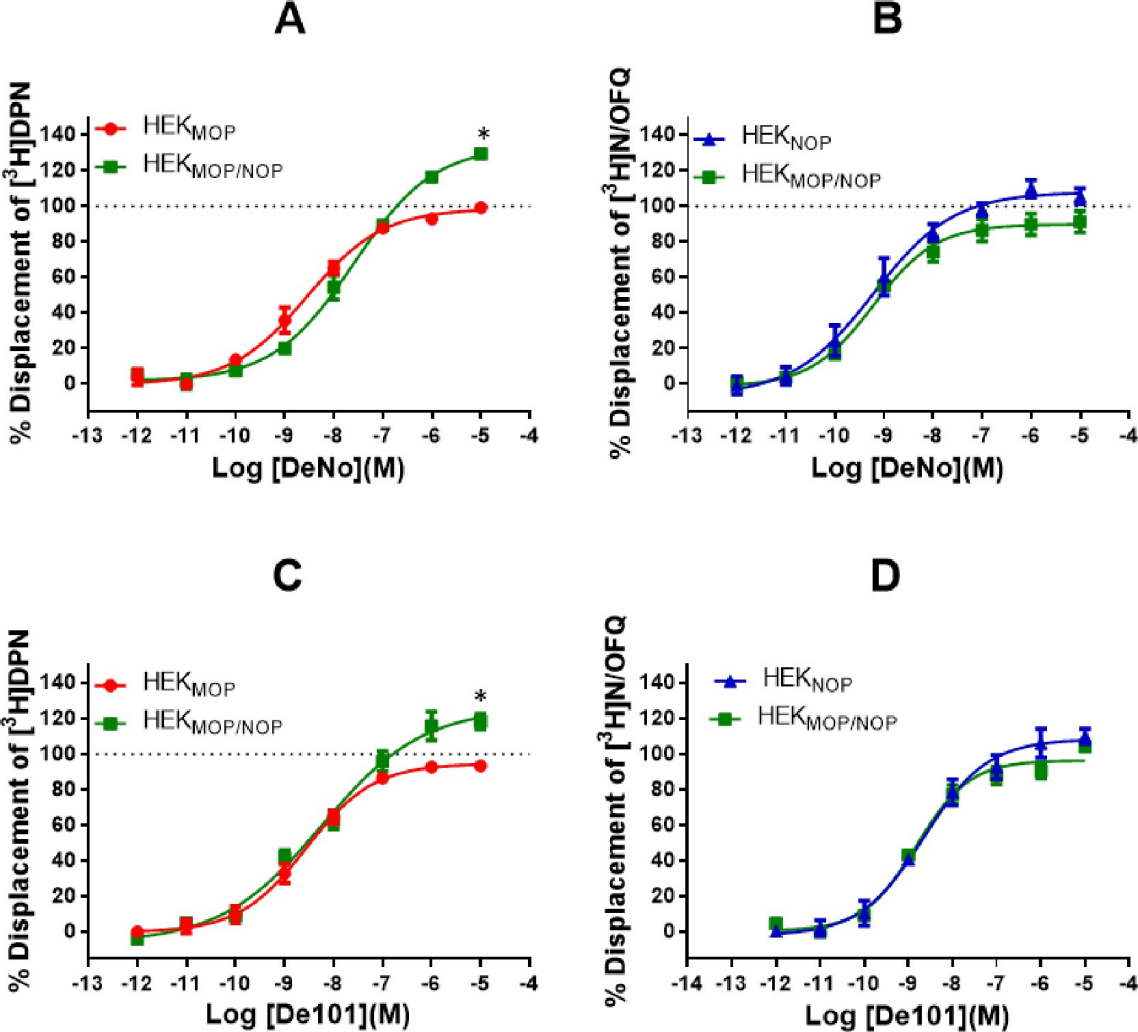
Displacement binding curves of A) DeNo in HEK_MOP_ and HEK_MOP/NOP_ cell membranes against [^3^H]-DPN or B) in HEK_NOP_ and HEK_MOP/NOP_ cell membranes against [^3^H]-N/OFQ. Displacement binding curves of C) De101 in HEK_MOP_ and HEK_MOP/NOP_ cell membranes against [^3^H]-DPN or D) in HEK_NOP_ and HEK_MOP/NOP_ cell membranes against [^3^H]-N/OFQ.Data are the mean (±SEM) of n = 5 experiments.

Both the bivalent ligands, DeNo and De101, displayed affinity across all three cell lines. In HEK_MOP_ cells, DeNo displaced [^3^H]-DPN with a pK_i_ of 9.00, DeNo demonstrated a statistically significant reduction in affinity for the MOP receptor in the co-expression system (pK_i_: 8.44) **(Fig 5 and Table 2)**. DeNo displaced [^3^H]-N/OFQ in both HEK_NOP_ and HEK_MOP/NOP_ cell lines with pK_i_ values of 9.69 and 9.59 respectively.

When using [^3^H]-DPN, De101 produced pK_i_ values of 9.28 and 8.63 for HEK_MOP_ and HEK_MOP/NOP_ respectively. De101 displaced [^3^H]-N/OFQ in HEK_NOP_ and HEK_MOP/NOP_, with pK_i_ values of 8.68 and 9.24 respectively **(Fig 5 and Table 2)**.

When using [^3^H]-DPN in HEK_MOP/NOP_ cell membranes, both DeNo (134±6%) and De101 produce displacement maxima in excess of 100% (126±3%; **Fig 5**), this was statistically significant compared to Dermorphin in HEK_MOP/NOP_ cells (p<0.05). This effect is not seen for these ligands in either of the single expression systems.

Collectively these data show that in the double expression system N/OFQ is able to displace [^3^H]-DPN and Dermorphin is able to displace [^3^H]-N/OFQ, further suggesting receptor interaction.

#### Confocal microscopy

**Fig 6:**
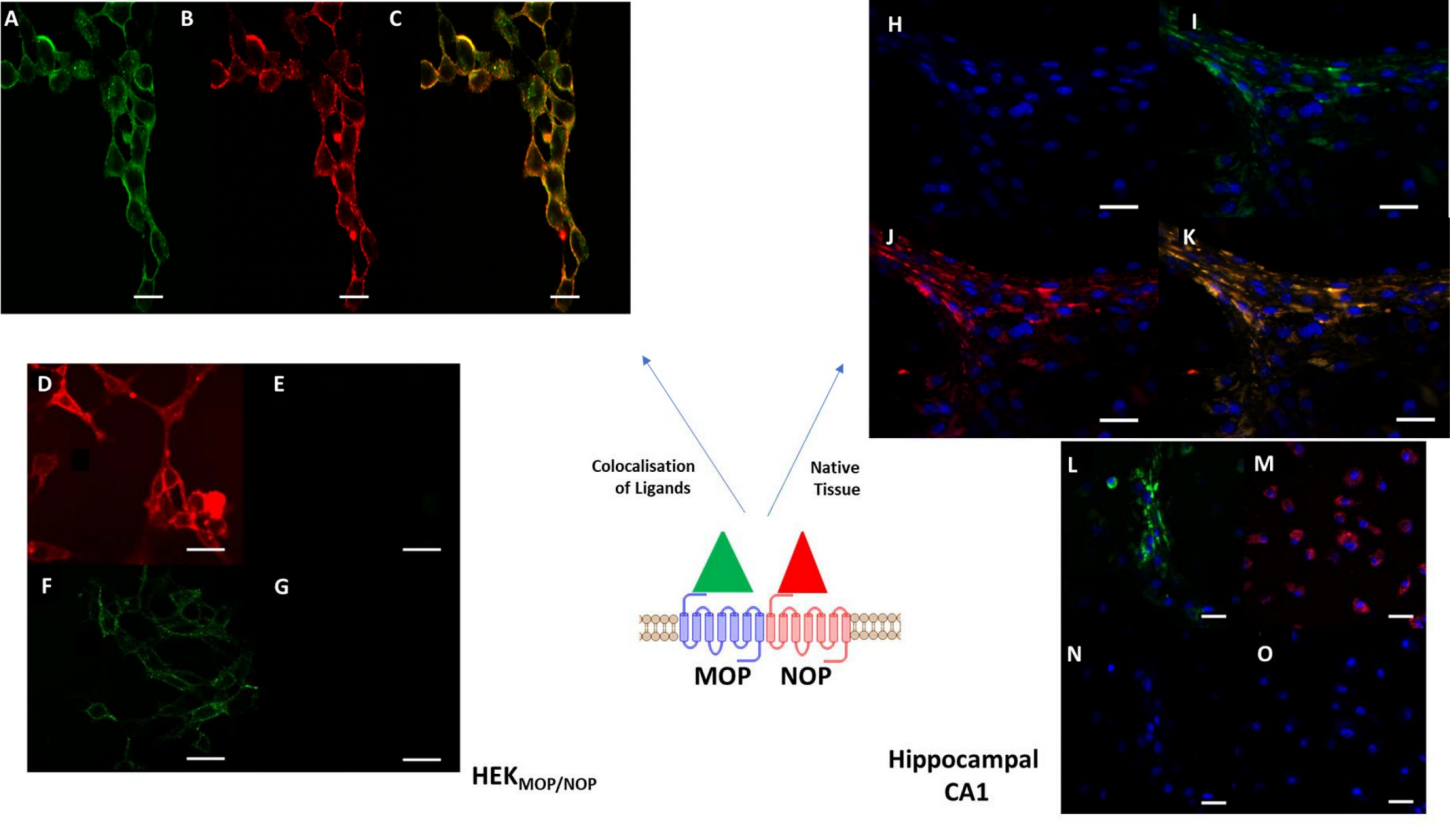
100nM of Derm_ATTO488_ (A) and 100nM N/OFQ_ATTO594_ (B) were co-incubated in HEK_MOP/NOP_ cells at 4°C, with both ligands binding to the cell surface. Image (C) demonstrates significant colocalisation (Pearson coefficient = 0.91). 100nM of Derm_ATTO488_ and 100nM of N/OFQ_ATTO594_ were added to HEK_MOP/NOP_ cells preincubated with 10μM naloxone and demonstrated binding of N/OFQ_ATTO594_ (D) but not Derm_ATTO594_ (E). 100nM of Derm_ATTO488_ and 100nM of N/OFQ_ATTO594_ were added to HEK_MOP/NOP_ cells preincubated with 10μM SB-612111 and demonstrated binding of Derm_ATTO488_ (F) but not N/OFQ_ATTO594_ (G). Mouse CA1 hippocampal cells were seeded onto 25mm glass coverslips, allowed to initiate process extension. Cells were treated with Ab-Delivirin ™ coated NeuN-alexaflour^405^ antibodies (Blue stained nucleus). Mouse hippocampal CA1 cells (H) were co-treated with 100nM each of Derm_ATTO488_ (I) and N/OFQ_ATTO594_ (J). The composite image (K) demonstrates significant co-localisation (Pearson coefficient = 0.83). In order to determine selectivity in mouse hippocampal CA1 tissue, a series of controls were undertaken in the presence of MOP (CTOP) and NOP (SB-612111) antagonists. In the presence of 10 μM SB-612111, 100nM Derm_ATTO488_ (L) bound to CA1 hippocampal cells, while the binding of N/OFQ_ATTO594_ was inhibited (N). 100nM Derm_ATTO488_ failed to bind to CA1 hippocampal cells after pretreatment with 10μM CTOP (O), however 100nM N/OFQ_ATTO594_ (M) retained binding affinity. All data are representative of 5 separate experiments. Scale bar (white) represents 20μm.

Co-localisation of MOP and NOP was determined through co-incubation with N/OFQ_ATTO594_ (NOP) and Dermorphin_ATTO488_ (MOP) (**Fig 6A–6C**). In HEK_MOP/NOP_ cells, binding of Dermorphin_ATTO488_ and N/OFQ_ATTO594_ was co-localized with a Pearson correlation coefficient of 0.91 suggesting that the two ligands, and hence receptors, were in close proximity to each other. Binding of N/OFQ_ATTO594_ in HEK_MOP/NOP_ was reversed in the presence of 10 μM SB-612111, but not the MOP antagonist Naloxone (10μM) (**Fig 6D and 6E)**. Binding of Derm_ATTO488_ was reversed in the presence of 10 μM Naloxone in HEK_MOP/NOP_, but not 10 μM SB-612111 (**Fig 6F and 6G)**.

In order to assess the potential for receptor interaction in non-recombinant systems, hippocampal (CA1) slices from mouse brain were incubated with 100 nM each of Dermorphin_ATTO488_ and N/OFQ_ATTO594_ **(Fig 6H–6K)**. Hippocampal slice tissue opioid receptor mRNA expression profile is shown in **S7 Table in S1 File**. The anti- NeuN antibody was used to select neuronal processes. Both fluorescent opioid ligands bound in similar regions (Pearson correlation coefficient 0.83), further suggesting receptor interaction in native tissue **(Fig 6K)**. Importantly, binding of Derm_ATTO488_ was fully inhibited by 10 μM of the MOP selective antagonist CTOP indicating the probe bound fully to MOP, while CTOP did not inhibit the binding of N/OFQ_ATTO594_ (**Fig 6M and 6O**). Administration of 10 μM of SB-612111 inhibited the binding of N/OFQ_ATTO594_, but not Derm_ATTO488_, again demonstrating selectivity for their respective receptors *ex vivo* (**Fig 6L and 6N**).

**Fig 7:**
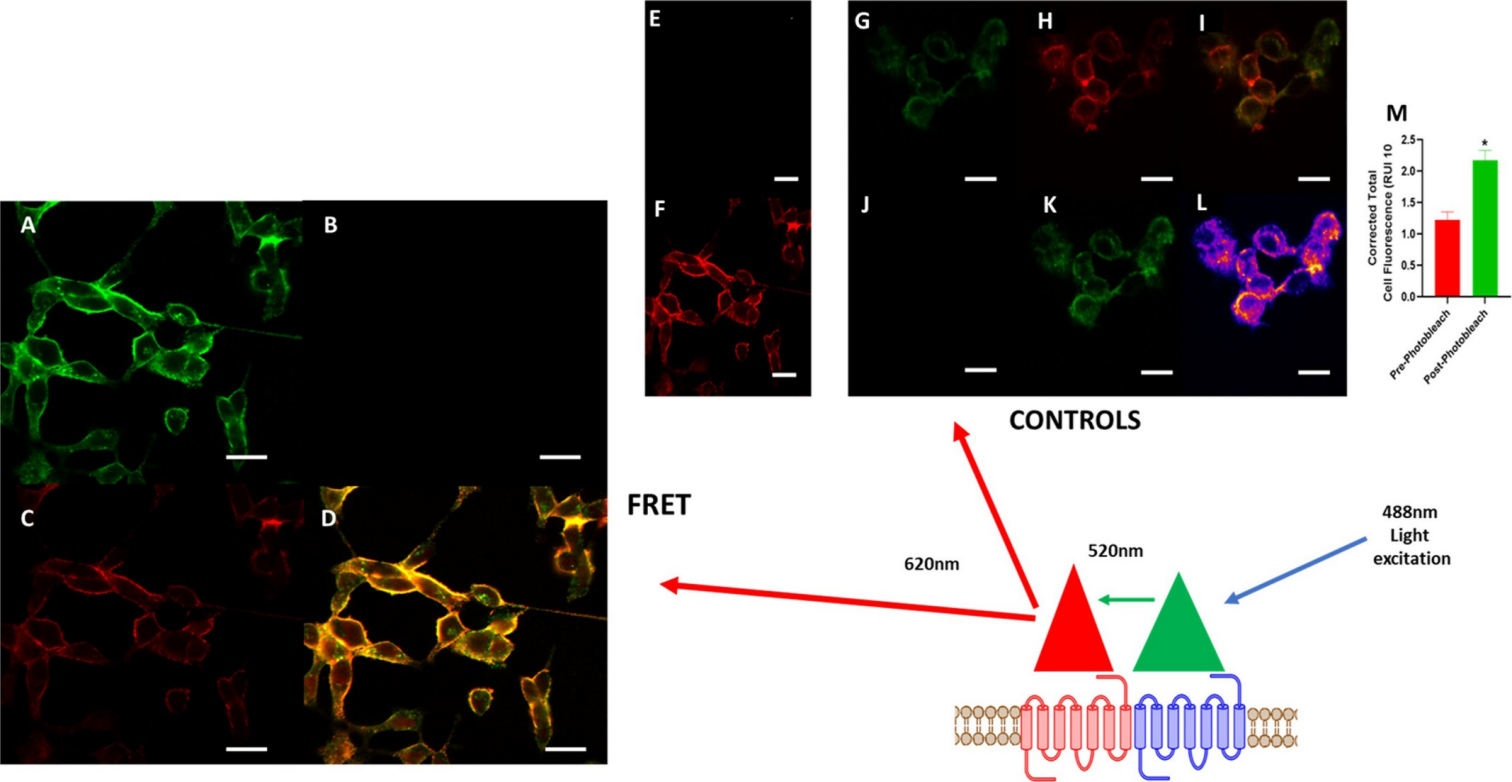
FRET was used to demonstrate proximity of the fluorescent ligands. (A) 100nM Derm_ATTO488_ bound to HEK_MOP/NOP_ cells is activated by 488nm laser and can be measured in the green channel; no fluorescence is demonstrated in the red channel (B). Following the addition of N/OFQ_ATTO594_, fluorescence is now detected by FRET in the red channel following stimulation with the 488nm laser (C). There is significant overlap with the green channel (D), shows merged image (Derm_ATTO_488 excited by 488nm wavelength, N/OFQ_ATTO594_ by 594nm wavelength) demonstrating colocalisation (Pearson Correlation Coefficient = 0.89) further indicating a close proximity between Derm_ATTO488_ and N/OFQ_ATTO594_. In order to demonstrate FRET-pairing of Derm_ATTO488_ and N/OFQ_ATTO594_, a series of control experiments were performed. N/OFQ_ATTO594_ (100nM) was incubated with HEK_MOP/NOP_ cells and stimulated by 488nm laser (E) and the 594nm laser (F). No fluorescence emission was demonstrated by stimulation with the 488nm laser, while N/OFQ_ATTO594_ was activated by the 594nm laser. A further confirmation of FRET pairing is through photobleaching of the acceptor molecule. HEK_MOP/NOP_ cells were co-incubated with 100nM Derm_ATTO488_ (G) and N/OFQ_ATTO594_ (H) with co-localisation demonstrated in (I). N/OFQ_ATTO594_ was exposed to 594nm until loss of fluorescence (i.e photobleaching) was seen (J). At this point, Derm_ATTO488_ fluorescence was measured (K) following which a heatmap (L) was generated, which demonstrated several areas of increased fluorescence (orange). The graph (M) demonstrates levels of fluorescence produced by Derm_ATTO488_ in HEK_MOP/NOP_ pre and post photobleaching (p<0.05; student’s t-test). Data are the mean±SEM of five experiment. Scale bar (white) represents 20μm.

To further demonstrate the close proximity of MOP and NOP in HEK_MOP/NOP_ cells, FRET experiments were performed using Derm_ATTO488_ and N/OFQ_ATTO594_ (**Fig 7A–7D**). Derm_ATTO488_ was incubated with HEK_MOP/NOP_ cells (100nM) and fluorescence was measured by 488 nm laser in both green (**A**) and red (**B**) filter channels. Fluorescence was detected in the green channel after stimulation by 488 nm laser, but not in the red channel. Subsequently, 100 nM N/OFQ_ATTO594_ was added and measured using the 488nm laser which does not activate N/OFQ_ATTO594_ alone. Fluorescence was detected in the red channel (**Fig 7C**), indicating FRET and therefore close proximity (**Fig 7D**) of Derm_ATTO488_ and N/OFQ_ATTO594_ and by inference of their receptors. **Fig 7E–7M** demonstrate the relevant controls to demonstrate FRET.

#### GTPγ[^35^S] assays

**Fig 8:**
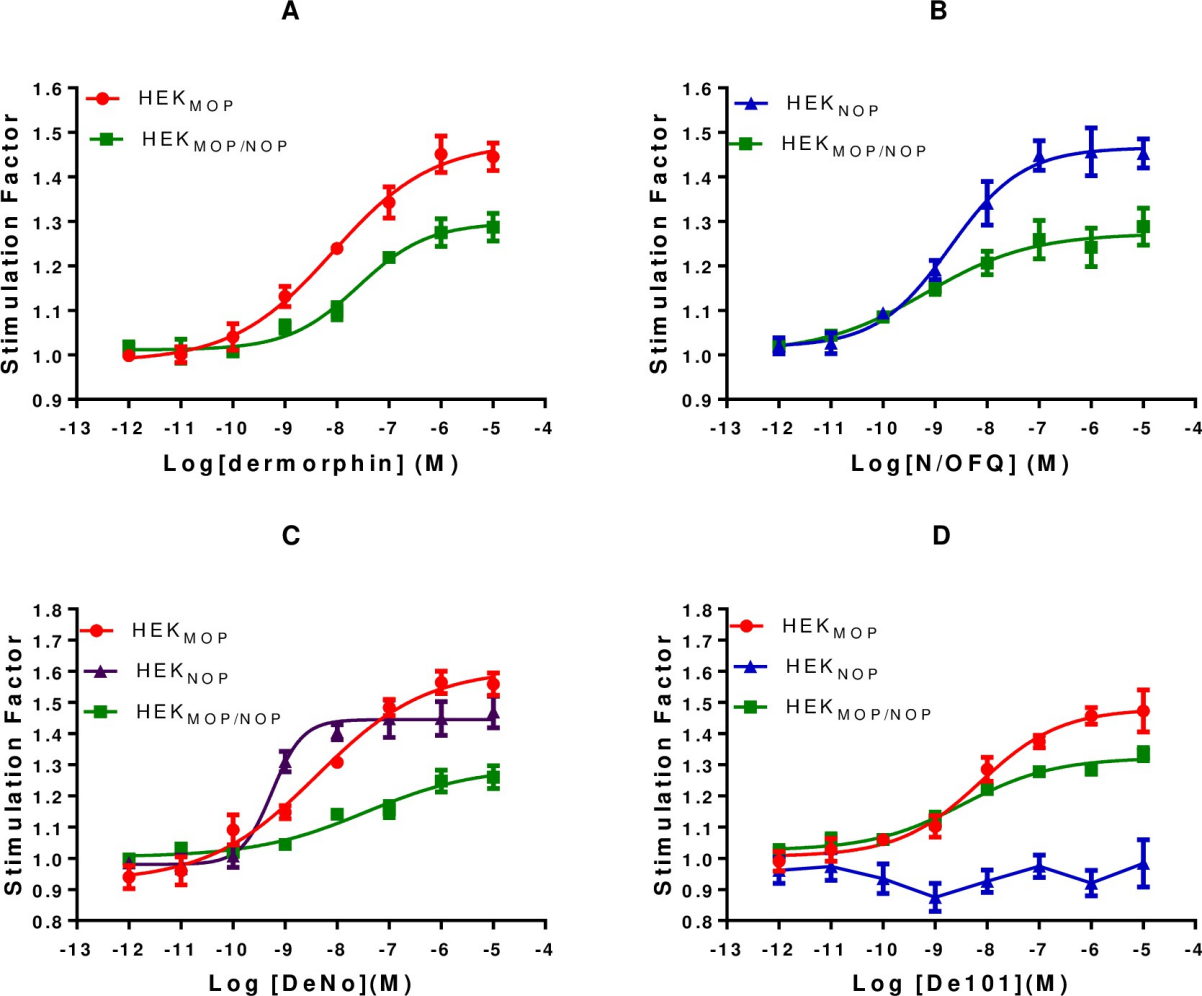
A) Dermorphin stimulated binding of GTPγ[^35^S] in HEK_MOP_ and HEK_MOP/NOP_ cell membranes. B) N/OFQ stimulated binding binding of GTPγ[^35^S] in HEK_NOP_ and HEK_MOP/NOP_ cell membranes. C) DeNO stimulated binding of GTPγ[^35^S] in HEK_MOP_, HEK_NOP_ and HEK_MOP/NOP_ cell membranes. D) De101 stimulated binding of GTPγ[^35^S] in HEK_MOP_, HEK_NOP_ and HEK_MOP/NOP_ cell membranes. Data are the mean (±SEM) for n = 5 experiments.

Dermorphin stimulated the binding of GTPγ[^35^S] in both HEK_MOP_ and HEK_MOP/NOP_ cell lines, with a statistically significant decrease in the pEC_50_ in the co-expression system **(Fig 8 and Table 3)**. N/OFQ stimulated a response in both HEK_NOP_ and HEK_MOP/NOP_ cell lines; in this case there was no significant change in agonist potency. DeNo produced a response in both MOP and NOP single expression and also in the co-expression system However, pEC_50_ values (pEC_50_:7.63; E_max_:1.26). in the co-expression system were significantly lower than those in both HEK_MOP_ and HEK_NOP_ cell membranes. The value for DeNO was not significantly different from that demonstrated by Dermorphin in HEK_MOP/NOP_. De101 produced a response in HEK_MOP_ cells but failed to produce a response in HEK_NOP_ cells. De101 produced a pK_b_ of 8.67±10 at 100 nM in antagonist assays when co-incubated with N/OFQ (**Fig 10**. De101 stimulated binding of GTPγ[^35^S] in HEK_MOP/NOP_ cell membranes producing a pEC_50_ of 8.67 and E_max_ of 1.34 **(Fig 8 and Table 3)**. De101 demonstrated a significant increase in pEC_50_ when compared to Dermorphin in the co-expression system. These data seem to indicate that where NOP is stimulated the response to MOP is shifted rightwards.

**Table 3:** Agonist stimulated GTPγS binding (left) and cyclic AMP inhibition (right-grey) in HEK_MOP_, HEK_NOP_ and HEK_MOP/NOP_.

	GTPγ[^35^S]						Cyclic AMP					
	HEK_MOP_		HEK_NOP_		HEK_MOP/NOP_		HEK_MOP_		HEK_NOP_		HEK_MOP/NOP_	
	pEC_50_	E_max_	pEC_50_	E_max_	pEC_50_	E_max_	pEC_50_	E_max_	pEC_50_	E_max_	pEC_50_	E_max_
Dermorphin	8.13_(±0.06)_	1.43_(±0.03)_	No Binding		7.60_(±0.07)_	1.31_(±0.04)_	8.81_(±0.06)_	75.59_(±3.28)_	No Activity		7.59_(±0.05)_	81.42_(±5.49)_
N/OFQ	No Binding		8.69_(±0.13)_	1.47_(±0.04)_	9.13_(±0.16)_^b^	1.25_(±0.05)_	No Activity		8.72_(±0.13)_	66.39_(±0.04)_	9.17_(±0.12)_^c^	86.35_(±4.28)_
DeNO	8.25_(±0.11)_	1.53_(±0.03)_	9.24_(±0.19)_^a^	1.47_(±0.05)_	7.63_(±0.22)_^b^	1.26_(±0.04)_	8.77_(±0.19)_	77.30_(±3.82)_	8.88_(±0.19)_	67.2_(±0.05)_	7.55_(±0.16)_	88.62_(±13.64)_
De101	8.26_(±0.11)_	1.44_(±0.03)_	No Activity		8.67_(±0.15)_^b^	1.34_(±0.05)_	9.00_(±0.11)_	73.28_(±2.63)_	No Activity		8.81_(±0.12)_^c^	85.99_(±8.82)_
Dermorphin & N/OFQ					7.70_(±0.29)_^,^^b^	1.28_(±0.03)_					8.04_(±0.22)_^c^	81.28_(±5.63)_
Dermorphin & UFP-101					8.46_(±0.14)_^b^	1.33_(±0.01)_					8.95_(±0.17)_^c^	84.42_(±3.30)_

^a^significant difference between the pEC_50_ of DeNO and De101 was found when compared to N/OFQ in HEK_NOP_ cells (p≤0.05);

^b^significant difference in potency when compared to the control ligand Dermorphin in HEK_MOP/NOP_ (p≤0.05).

^c^ significant difference in potency when compared to the control ligand Dermorphin in HEK_MOP/NOP_ (p≤0.05). Data are displayed as mean (±SEM) of n = 5 experiments. p≤0.05 (ANOVA) followed by post hoc Bonferroni multiple comparisons.

It has previously been reported that linker length in bivalent pharmacophores can have an effect on receptor binding leading to changes in potency and efficacy. In order to determine whether such effects were occurring in DeNo and De101, Dermorphin and N/OFQ were co-incubated (as individual unlinked peptides) in the co-expression system. This combination of ligands produced a response (pEC_50_:7.70; E_max_:1.28), which was not significantly different from the pEC_50_ for DeNo in the co-expression system, but was significantly different from their respective pEC_50_ values in single expression systems **(S7A Fig in S1 File and Table 3)**. Co-incubation of Dermorphin and UFP-101 (pEC_50_: 8.46; E_max_:1.33) produced a response similar to De101 in the co-expression system. These data suggest that linkage *per se* was unimportant.

#### Cyclic Adenosine Monophosphate (cAMP) assay

**Fig 9:**
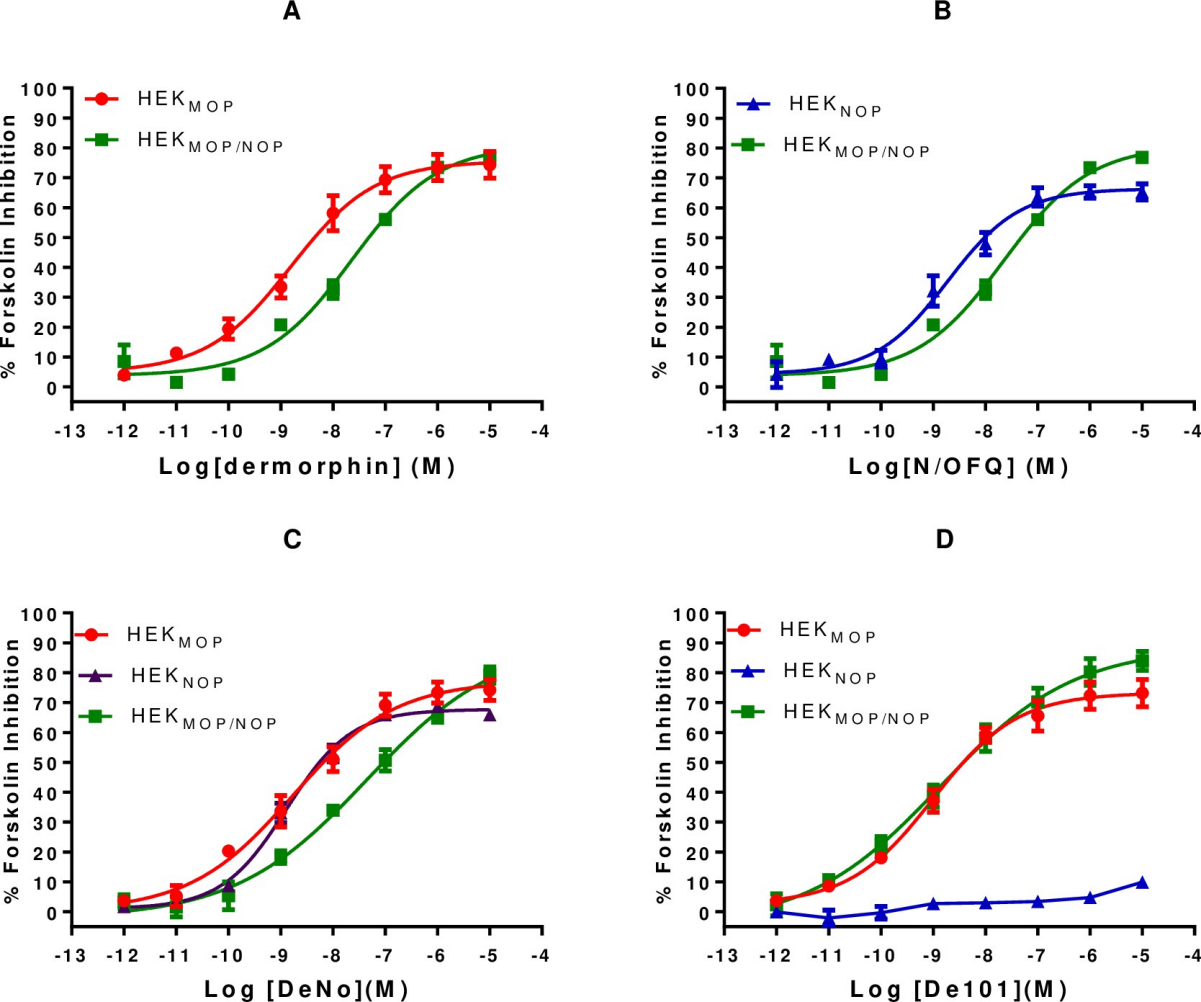
A) Dermorphin stimulated inhibition of forskolin-induced cAMP production in HEK_MOP_ and HEK_MOP/NOP_ cells. B) N/OFQ stimulated inhibition of forskolin-induced cAMP production in HEK_NOP_ and HEK_MOP/NOP_ cells. C) DeNO stimulated inhibition of forskolin-induced cAMP production in HEK_MOP_, HEK_NOP_ and HEK_MOP/NOP_ cells. D) De101 stimulated inhibition of forskolin-induced cAMP production in HEK_MOP_, HEK_NOP_ and HEK_MOP/NOP_ cells. Data are the mean (±SEM) for n = 5 experiments.

cAMP inhibition assays demonstrated a similar trend in function for both the monovalent and bivalent ligands. Dermorphin produced a concentration-dependent inhibition of forskolin-stimulated cAMP formation in HEK_MOP_ and HEK_MOP/NOP_ whole cells. Dermorphin pEC_50_ was significantly reduced in the co-expression system **(Table 3 and Fig 9)**. N/OFQ produced concentration-dependent inhibition of forskolin stimulated cAMP formation in HEK_NOP_ and HEK_MOP/NOP_ cells. There was no significant difference in the values obtained for N/OFQ in both cell lines **(Table 3 and Fig 9)**.

In HEK_MOP_, HEK_NOP_ and HEK_MOP/NOP_ cells, DeNO produced a concentration-dependent inhibition of forskolin stimulated cAMP formation. The pEC_50_ value obtained by DeNo in HEK_MOP/NOP_ cells was significantly lower than both values obtained in HEK_MOP_ or HEK_NOP_ cells **(Table 3 and Fig 9)**. De101 produced a concentration-dependent inhibition of forskolin stimulated cAMP formation in HEK_MOP_ and HEK_MOP/NOP_ cells while being inactive in HEK_NOP_ cells. In HEK_MOP/NOP_ cells, De101 produced a pEC_50_ of 8.81 and E_max_ of 85.99%, which was significantly higher than Dermorphin in this cell line. **(Table 3 and Fig 9)**.

Further to the experiments conducted in GTPγ[^35^S] assays to determine whether linkage of pharmacophores could influence activity, Dermorphin and N/OFQ or Dermorphin and UFP-101 were co-administered over a range of concentrations in HEK_MOP/NOP_ cells **(S7B Fig in S1 File and Table 3)**. Dermorphin and N/OFQ produced a pEC_50_ of 8.04, similar to both Dermorphin alone, or DeNO in the co-expression system but significantly lower than N/OFQ alone in HEK_MOP/NOP_ and HEK_NOP_ **(Table 3)**. Dermorphin co-administered with UFP-101 produced a pEC_50_ of 8.95, which was significantly greater than Dermorphin in the co-expression system, but similar to the potency of Dermorphin in HEK_MOP_ cell line **(Table 3)**.

**Fig 10:**
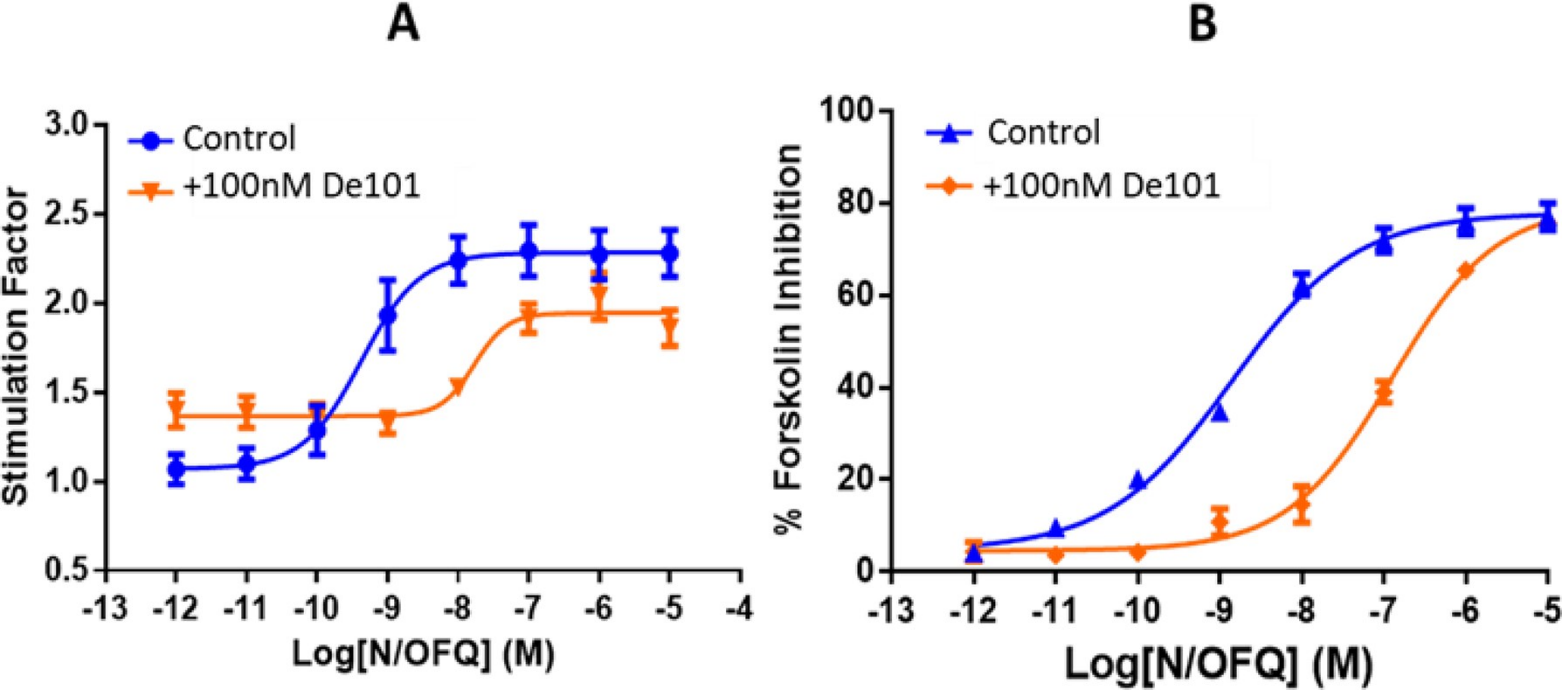
Demonstration of antagonist activity of De101 (100nM) against a range of concentrations of N/OFQ in (A) GTPγ[^35^S] assays in CHO_NOP_ and (B) in HEK_NOP_ cells using a cyclic AMP assay. Data are the mean (±SEM) of n = 5 experiments.

A further demonstration of De101 antagonist activity was measured in the cyclic AMP inhibition assay. In these experiments De101 produced a pK_b_ of 8.77 (±0.18) (**Fig 10B**).

As in GTPγ[^35^S] assays, in the co-expression system NOP activation led to a rightward shift in the curve of MOP agonists. The results would indicate activation of NOP leads to an inhibitory action on MOP receptor ligand activation when these two receptors are co-expressed.

#### ERK1/2 activity

**Fig 11:**
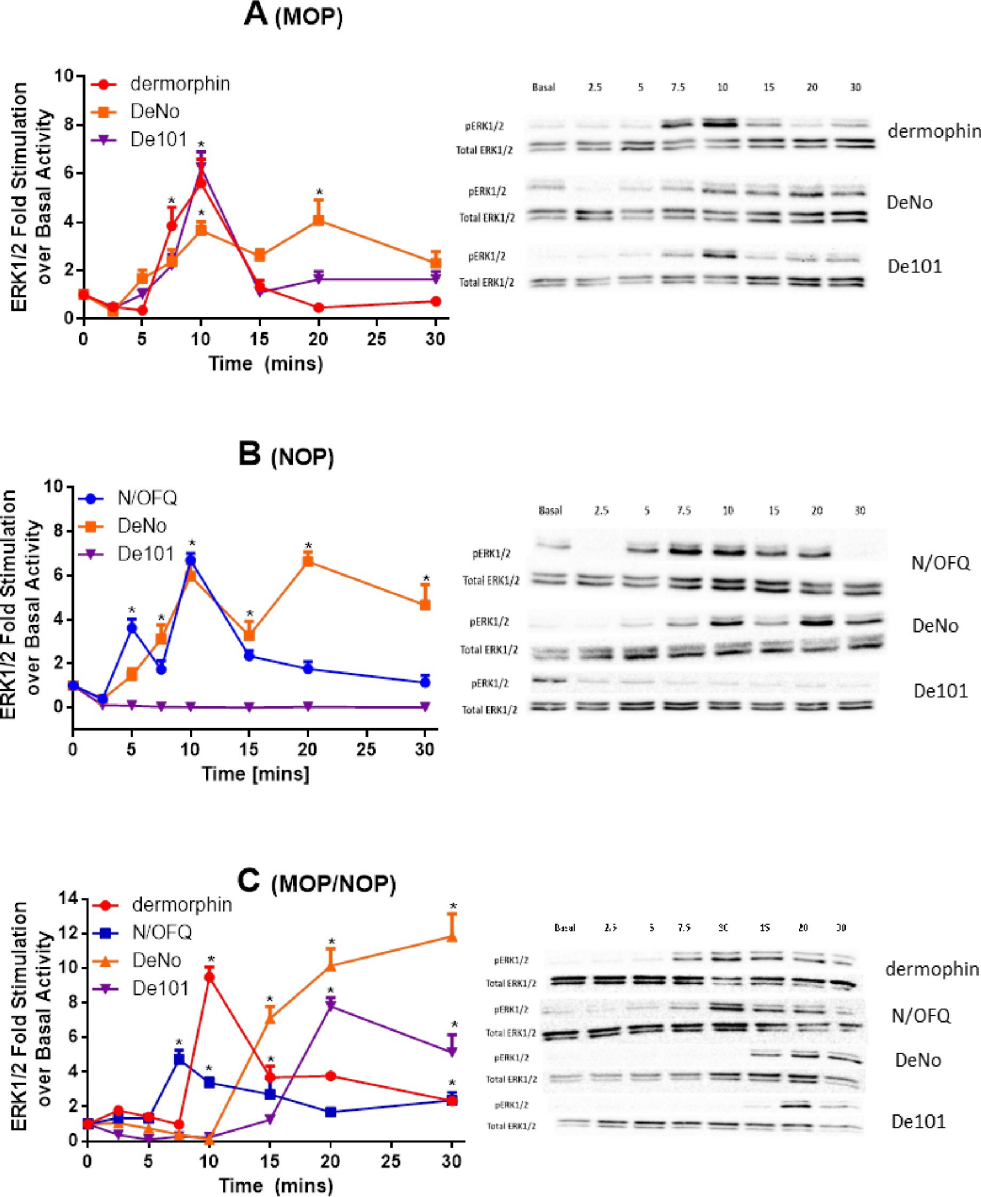
(A) ERK1/2 phosphorylation in HEK_MOP_ cell lines induced by Dermorphin, DeNo and De101. (B) N/OFQ, DeNO and De101 induced ERK1/2 phosphorylation in HEK_NOP_. (C) The induction of ERK1/2 phosphorylation produced by Dermorphin, N/OFQ, DeNo or De101 in HEK_MOP/NOP_ cell lines. Right hand panels are representative. In HEK_MOP,_ Dermorphin (F_(1.14,4.57)_ = 26.59; p<0.0042), DeNO (F_(7,32)_ = 8.37; p<0.0001) and De101 (F_(7,32)_ = 17.55; p<0.0001) were significant. In HEK_NOP_, N/OFQ (F_(7,32)_ = 16.91; p<0.0001) and DeNO (F_(7,32)_ = 10.11; p<0.0001) were significant while De101 (F_(7,32)_ = 1; p = 0.45) was not. In HEK_MOP/NOP_, Dermophin (F_(7,32)_ = 8.93; p<0.0001), N/OFQ (F_(7,32)_ = 7.43; p<0.0001), DeNO (F_(7,32)_ = 20.22; p<0.001) and De101 (F_(7,32)_ = 19.53; p<0.001) were all significant. *significant increase in activity when compared to basal. p≤0.05 (ANOVA) followed by post hoc Bonferroni multiple comparisons. Data are the mean (±SEM) for n = 5 experiments.

Dermorphin (1 μM) produced a time-dependent increase in pERK1/2 in both HEK_MOP_ and HEK_MOP/NOP_ cells **(Fig 11)**. In HEK_MOP_ cells this peaked at 10 min (maximum fold phosphorylation 7.21), which subsequently returned to basal levels after 15 min. In HEK_MOP/NOP_ cells, Dermorphin peaked at 10 minutes (maximum fold phosphorylation 11.82) and remained elevated for the duration of the time course (30 min).

N/OFQ (1 μM) produced a time-dependent increase in pERK1/2 in both HEK_NOP_ and HEK_MOP/NOP_ cells **(Fig 11)**. In HEK_NOP_ cells, N/OFQ produced a biphasic stimulation of ERK1/2 activity which peaked at 5 (3.91 Fold) and 10 min (5.89 fold), and returned to basal after 15 min. In HEK_MOP/NOP_ cells, N/OFQ-stimulated pERK1/2 activity reached a peak at 7.5–10 min (~5 fold). ERK1/2 activity reduced from 15 min and remained above basal activity for the duration of the time course.

In HEK_MOP_, HEK_NOP_ and HEK_MOP/NOP_ cells, DeNO (1μM) produced a time-dependent increase in pERK1/2 **(Fig 11)**. In HEK_MOP_ cells, DeNO produced a slow phosphorylation of ERK1/2 peaking around 10 min (3.65 fold) and remained elevated. In HEK_NOP_ cells, DeNo produced a biphasic increase at 10 (8.16 fold) and 20 min (10.03 fold). In HEK_MOP/NOP_ cells, the phosphorylation of ERK1/2 was delayed beginning at 15 min and remain elevated at 30 min (11.84 fold).

In HEK_MOP_ and HEK_MOP/NOP_ cells, De101 (1μM) produced a time-dependent increase in pERK1/2 **(Fig 11)**. In HEK_MOP_ cells, De101 produced a peak phosphorylation of ERK1/2 at 10 min (9.65 fold), followed by a prompt decline to basal. In HEK_MOP/NOP_ cells, the phosphorylation of ERK1/2 was delayed beginning at 20 min (12.59 fold) and remaining elevated at 30 min. De101 did not phosphorylate of ERK1/2 in HEK_NOP_ cells.

Full uncropped blots are available as a supplementary document.

## Discussion

We have generated a MOP/NOP co-expression system which we have used to determine (i) how targeting of two opioid receptors affects cellular signalling cascades and (ii) evidence for receptor interaction. Our HEK_MOP/NOP_ expressed similar numbers of NOP (689 fmol/mg protein) and MOP (464 fmol/mg protein) receptors. Similar levels of expression of the two receptors of interest is important such that the potential to create receptor and coupling reserves is equal.

Using our novel fluorescent ligands [14,39], we aimed to determine whether MOP and NOP receptors were expressed in close proximity to each other in our co-expression system. A significant overlap in binding of both fluorescent probes on the HEK_MOP/NOP_ cell surface was detected, producing a Pearson coefficient correlation of 0.91 (High level of colocalization). Furthermore, FRET experiments demonstrate the close proximity of the fluorescent ligands and, by extension, MOP and NOP receptors. For FRET to occur ligands must be within 10 nm of each other [54] thereby indicating that these two receptors are close enough to potentially form a structural interaction [55]. However, this is not a natural system with both receptors expressed due to cloning techniques. In order to demonstrate the potential for co-expression in a native system, we assessed the binding of N/OFQ_ATTO594_ and Derm_ATTO488_ in *ex vivo* CA1 hippocampal neuronal processes. The NOP selective antagonist SB-612111 and the MOP-selective antagonist CTOP were used to demonstrate selectivity of binding of the fluorescent ligands in the native system and, fluorescent antibodies to NeuN were used to identify neuronal outgrowths. A Pearson coefficient correlation of 0.83 again indicates a high level of co-expression and, therefore, co-expression in the same cell. As a reminder and a note of caution with these experiments; the ligands are both agonists so to prevent internalisation imaging was performed at 4°C and this may limit receptor movement in the membrane. If there is a constitutive interaction then there are no issues but if an interaction is ligand driven low temperature may reduce or slow this interaction. Experiments using other methods to reduce internalisation such as high sucrose could address this issue. Taken as a whole this data provides evidence of MOP/NOP interaction also in native tissue.

Primary pharmacological support for the cellular interaction of MOP and NOP comes from the observation that Dermorphin was able to displace [^3^H]-N/OFQ in the co-expression system, and conversely N/OFQ was able to displace [^3^H]-DPN, characteristics not demonstrated by these ligands in single expression systems. Moreover, all ligands tested demonstrated a loss of affinity at the MOP receptor in the co-expression system.

The ability of high affinity MOP receptor ligands to displace [^3^H]-N/OFQ has been demonstrated previously [34]. However, to the best of our knowledge the effect of NOP ligands on MOP receptor binding has not been previously demonstrated. In this paper, we show that displacement of radioligand binding by MOP or NOP selective ligands (as demonstrated in single receptor systems) is bidirectional in the co-expression system. Both Dermorphin and N/OFQ fail to produce 100% displacement of these respective radioligands in HEK_MOP/NOP_ cell membranes. For both DeNo and De101, we demonstrate that these bivalent ligands are able to displace 140% and 120% of [^3^H]-DPN in HEK_MOP/NOP_ membranes, which is significantly higher than that of the single pharmacophore ligands. This effect is not seen with in [^3^H]-N/OFQ displacement assays. We have no obvious explanation for this phenomenon but modification of the binding pocket(s) in a potential dimeric conformation might modify the way bivalent ligands interact.

If drugs are to be developed targeting the MOP/NOP heterodimer it is essential to understand any potential changes in downstream signalling. The first pathway investigated in this study was the initial G-protein activation, through measurement of GTPγ^35^S binding. The first evidence for changes in signalling due to direct interaction of MOP and NOP is seen with the peptide Dermorphin, with a loss of potency in the co-expression system when compared to the HEK_MOP_ single-expression system. This is comparable with loss of potency seen in DAMGO by Wang and colleagues (2005) in their MOP/NOP co-expression system [35]. There is limited research to determine the effects of co-administration of MOP and NOP ligands, or drugs developed to target both receptors simultaneously, an area this study seeks to address. From the perspective of the NOP receptor, N/OFQ potency increases in the co-expression system. Of more interest is the activity of the bivalent pharmacophore, DeNO. DeNo demonstrates a significant loss of potency when compared to results in both HEK_MOP_ and HEK_NOP_. DeNo demonstrated similar potencies to Dermorphin in previous studies, so to determine whether the loss of potency was as a result of cellular interaction of MOP and NOP, Dermorphin and N/OFQ were administered in HEK_MOP/NOP_ cells. The results obtained demonstrated a similar potency for Dermorphin in the co-expression system (significantly lower than in single expression system), but also demonstrated a loss of potency for N/OFQ when co-administered with Dermorphin. The MOP agonist-NOP antagonist ligand De101, demonstrated no changes in potency when administered in the co-expression system when compared to single expression systems. Furthermore, the co-administration of Dermorphin and the NOP antagonist UFP-101 produced a potency similar to that produced in HEK_MOP_ cells by Dermorphin alone.

The results seen in GTPγ[^35^S] functional assays were mirrored by those seen in cAMP assays. Dermorphin, DeNO and a combination of Dermorphin and N/OFQ all demonstrated a reduction in potency in HEK_MOP/NOP_ cells. N/OFQ, administered alone, produced a higher potency in the co-expression system, while De101 demonstrated unchanged potency values in the co-expression system when compared to HEK_MOP_. These results further support the suggestion of a MOP/NOP heterodimer that, when formed, leads to changes in signalling.

The final signalling pathway investigated was the ERK1/2 MAPK pathway, activated by both canonical (G-protein) and non-canonical signalling (Arrestin) pathways. The ERK1/2 pathway is involved in numerous cellular functions including proliferation, transcription activation and cell death, all of which are controlled by spatio-temporal activation of the protein itself, with activation at MOP beginning around 2–5 minutes post drug administration peaking from 7.5–10 minutes post administration [56]. The bivalent pharmacophores, DeNo and De101, both demonstrated a significant delay in activation of ERK1/2 in the HEK_MOP/NOP_ line when compared to Dermorphin and N/OFQ. More interestingly, the peak activation of ERK1/2 by DeNO occurred at the latest time point in our study. The results again suggest the interaction of MOP and NOP may lead to significant changes in opioid ligand signalling, whether this be through structural interaction or changes in recruitment of G-protein receptor kinase (GRK) and arrestin requires further investigation. As previously demonstrated by Hawes and colleagues, both NOP and MOP activate MAPK via G_i_ beta/gamma. Moreover, in CHO cells expressing both receptors pre-treatment with N/OFQ reduced NOP and MOP activation (with DAMGO) of MAPK. These data indicate NOP is modulating MOP signalling [57].

While we provide strong evidence (structural and functional) for direct interaction between MOP and NOP, we cannot confirm heterodimerisation. The overall effect of targeting both MOP and NOP simultaneously appears to be negative with respect to MOP. An important question arising from these findings is does this negative effect carry forward to physiological action with regards to analgesia? The results provided by mixed MOP/NOP agonists such as buprenorphine [16] and cebranopadol [17] wouold suggest that the overall effect is beneficial, as these drugs d display analgesia with reduced side-effects. That said this may be due to targeting MOP and NOP on different cells/pathways or on the same cell if structural receptor interaction is a driver. Future work could include disruption of the heterodimer using single transmembrane domains such as the example used by He and colleagues, whereby the introduction of MOR^TM1^-TAT lead to the disruption of the MOP-DOP heterodimer [58].

There are several potential limitations to our work probing MOP and NOP interactions. Firstly, the use of transfected cells which can produce significantly higher numbers of receptors than seen native tissue; we have tried to control for this by selecting clones with similar levels of expression for MOP and NOP in single expression systems and both MOP/NOP in the double expression system. Expression differences/overexpression could lead to ‘forced’ interactions between the receptors, to manifest as co-localisation or changes in signalling pathways due to receptor competition for G proteins and or GRKs. In order to assess whether the close proximity of MOP and NOP is due simply to high expression, we extended our study to include co-expression in native tissue and provide significant evidence that interaction of the type seen in recombinants also occurs in a native tissue; mouse hippocampal neurites. Secondly, signalling differences seen in the co-expression system such as reduction in MOP agonist potency may be due to either competition at downstream signalling pathways or, due to the decrease in receptor expression of MOP in the co-expression system when compared to single expression system, a lack of receptor reserves (this could potentially be probed for MOP with β-funaltrexamine) and therefore decreased potency. We believe this is not the case, as both De101 and dermorphin co-incubated with UFP-101 demonstrate similar or higher potency to the single expression MOP system. If receptor competition for downstream signalling pathways was occurring, this reversal would only be possible if UFP-101 was acting as an inverse agonist, for which we have no evidence.

What does this data mean for development of bivalent opioids and, by extension, bifunctional opioids? Work with cebranopadol and other mixed opioids is now maturing and, in general, these produce good analgesia with reduced side effects. MOP-morphine like molecules are currently seen as the ‘enemy’; driven by the opioid epidemic. That said MOP analgesia and analgesics used in the right setting is/are good [59]. Attempts to design out the troublesome side effect profile have been met with variable success so a different approach with mixed ligands is worthy of consideration. A mixed ligand (bivalent or bifunctional) can potentially reduce the ‘amount’ of MOP activation by adding in NOP (or other targets) with the NOP component producing analgesia in its own right along with reducing the adverse effects of MOP. There is an excellent study in non-human primates from Ko and Naughton [24] showing that largely ineffective doses of i.t morphine and N/OFQ synergise to produce good quality antinociception. Add into the mix partial agonists/biased agonists (for example oliceridine) and allosteric modulators and the potential for analgesic design increases [60,61].

## Supporting information

S1 File (DOCX)

S1 Raw images (PDF)
