# Supplementary material for: MOP and NOP receptor interaction: Studies with a dual expression system and bivalent peptide ligands
Source: PLoS One. 2022 Jan 21;17(1):e0260880. doi: 10.1371/journal.pone.0260880 (PMC8782398; doi:10.1371/journal.pone.0260880)
Supplement: S1 File — (DOCX) [file pone.0260880.s001.docx]

# MOP AND NOP RECEPTOR INTERACTION: STUDIES WITH A DUAL EXPRESSION SYSTEM AND BIVALENT PEPTIDE LIGANDS.

# SUPPLEMENT.

M.F Bird^1^, J McDonald^1^, B Horley^1^, J.P O’Doherty^1^, B. Fraser^2^. C.L. Gibson^3^, R Guerrini^4^, G Caló^5^, D.G Lambert^1^

^1^Department of Cardiovascular Sciences, University of Leicester, Anaesthesia, Critical Care and Pain Management, Leicester Royal Infirmary, Leicester, LE2 7LX. UK.

^2^Department of Neuroscience, Psychology and Behaviour, University of Leicester, Henry Wellcome Building, LE17HB, UK.

^3^School of Psychology, University of Nottingham, Psychology Building, University Park, NG7 2RD, UK.

^4^Department of Chemical, Pharmaceutical and Agricultural Sciences, University of Ferrara, 44121 Ferrara, Italy.

^5^Department of Pharmaceutical and Pharmacological Sciences, University of Padova, 35131 Padova, Italy.

**1. Cloning and Selection of MOP and NOP receptor expressing cell lines**

HEK_W/T293-A_ were transfected with either MOP plasmid (geneticin selectivity) or NOP plasmid (Hygromycin B selectivity) using FugeneHD® transfection reagent (Promega, UK). Both plasmids were purchased from cDNA.org. Following transfection, transfected cells were exposed to high dosing of the requisite antibiotic (MOP- 1000ug.ml^-1^ Geneticin; NOP- 800ug.ml^-1^ Hygromycin B). Clones which survived this initial process were subject to single cell dilution and colonies from individual cells were grown as clone colonies. When colonies reached confluence, they were screened for the presence of MOP or NOP mRNA (**MOP:Table S1 and NOP Table S3).** Clones found to have a high Δ Ct (HK gene GAPDH) were tested in radioligand binding saturation experiments to measure surface receptor expression using either [^3^H]-DPN (**Figure S1, Table S2**) or [^3^H]-N/OFQ (**Figure S2, Table S4**) [1]. Clones demonstrating high expression of the relevant receptor were retained and measured after 10 passages to assess receptor expression and stability (**Table S2&S4**). HEK_MOP_ clone #1(pK_d_:9.26; B_max_:1603) and HEK_NOP_ clone #8(pK_d_:8.93; B_max_:887) were shown to have the highest and most stable expression. HEK_MOP_ clone #1 was transfected with NOP plasmid and selection process was undertaken using Geneticin and Hygromycin B. Surviving clones were screened using qPCR to assess both MOP and NOP mRNA expression. Clones showing high levels of mRNA (**Table S5**) for both receptors were tested in saturation binding assays using [^3^H]-DPN (or [^3^H]-N/OFQ (**Figure S3, Table S6**).

| Clone | GAPDH C_t_ | MOP C_t_ | Δ C_t_ Mean |
| --- | --- | --- | --- |
| HEK_MOP_ #1 | 20.21 | 19.58 | -0.62 |
| HEK_MOP_ #2 | 20.49 | 20.57 | 0.08 |
| HEK_MOP_ #3 | 19.89 | 24.68 | 4.79 |
| HEK_MOP_ #4 | 19.56 | 20.01 | 0.45 |
| HEK_MOP_ #5 | 18.85 | 18.31 | -0.55 |
| HEK_MOP_ #6 | 19.62 | 36.43 | 16.81 |
| HEK_MOP_ #7 | 20.73 | 19.78 | -0.95 |
| HEK_MOP_ #8 | 19.83 | 20.08 | 0.25 |
| HEK_MOP_ #9 | 21.75 | 20.76 | -0.98 |
| HEK_MOP_ #10 | 19.45 | 28.53 | 9.07 |
|  |  |  |  |

**Table S1**: C_t_ values for HEK_MOP_ clones. Clones displaying alow ΔC_t_ values are shaded grey. Samples were screened from single cell RNA extractions (n=1).


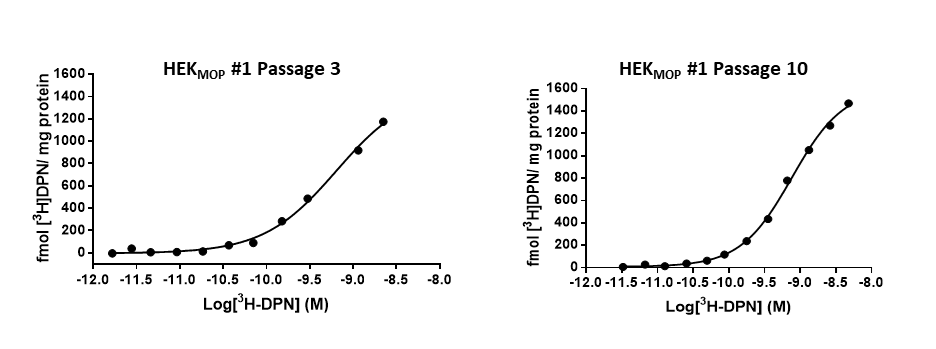


**Figure S1**: Representative [^3^H]-DPN saturation binding curves depicting receptor expression in HEK_MOP_ #1 clones over a window of 7 passages.

|  | Passage 3 | | Passage 10 | |  |
| --- | --- | --- | --- | --- | --- |
|  | pK_d_ | B_max_ | pK_d_ | B_max_ |  |
| **HEK_MOP_ #1** | **9.23** | **1542** | **9.26** | **1603** |  |
| HEK_MOP_ #2 | 9.26 | 1325 | 9.23 | 850 |  |
| HEK_MOP_ #4 | 9.28 | 1125 |  |  |  |
| HEK_MOP_ #5 | 9.19 | 863 |  |  |  |
| HEK_MOP_ #7 | 9.22 | 534 | 9.32 | 263 |  |
| HEK_MOP_ #8 | 9.25 | 1432 |  |  |  |
| HEK_MOP_ #9 | 9.23 | 723 | 9.28 | 605 |  |

**Table S2**: The B_max_ and pK_d_ values obtained from saturation binding assays using [^3^H]-DPN with HEK_MOP_ monoclonal cell lines, over several passages. Areas shaded in dark, indicate that the cell died between passages. The rate of cell growth was also monitored, and aided the cell line choice for future experiments.

| Clone | GAPDH C_t_ | NOP C_t_ | Δ C_t_ Mean |
| --- | --- | --- | --- |
| HEK_NOP_ #2 | 21.87 | 31.23 | 9.35 |
| HEK_NOP_ #3 | 21.53 | 30.77 | 9.24 |
| HEK_NOP_ #4 | 21.81 | 31.23 | 9.43 |
| HEK_NOP_ #8 | 21.38 | 20.22 | -1.16 |
| HEK_NOP_ #9 | 22.74 | 21.29 | -1.46 |
| HEK_NOP_ #13 | 21.16 | 19.93 | -1.23 |
| HEK_NOP_ #16 | 22.47 | 20.75 | -1.72 |
| HEK_NOP_ #17 | 21.20 | 19.88 | -1.32 |
| HEK_NOP_ #19 | 22.37 | 21.37 | -1.00 |
| HEK_NOP_ #23 | 21.40 | 20.12 | -1.28 |
| HEK_NOP_ #25 | 31.84 | 29.73 | -2.11 |
|  |  |  |  |

**Table S3**: C_t_ values for HEK_NOP_ clones. Samples were screened from single cell RNA extractions (n=1).


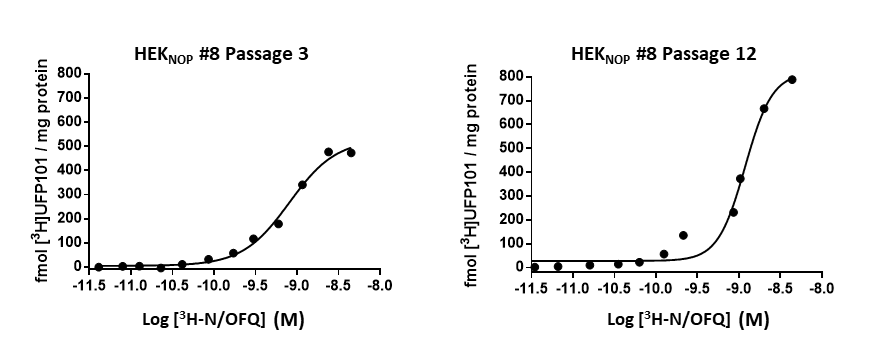


**Figure S2**: Representative [^3^H]-N/OFQ saturation binding graphs depicting receptor expression in HEK_NOP_ #1 clones over a window of 9 passages.

|  | Passage 3 | | Passage 10 | |
| --- | --- | --- | --- | --- |
|  | pK_d_ | B_max_ | pK_d_ | B_max_ |
| HEK_NOP_ #8 | 9.09 | 529.4 | 8.93 | 887.0 |
| HEK_NOP_ #9 |  |  |  |  |
| HEK_NOP_ #13 |  |  |  |  |
| HEK_NOP_ #16 | 9 | 432.5 | 9.01 | 482.3 |
| HEK_NOP_ #17 | 8.99 | 270.3 |  |  |
| HEK_NOP_ #19 |  |  |  |  |
| HEK_NOP_ #23 |  |  |  |  |
| HEK_NOP_ #25 | 9.10 | 352.3 |  |  |

**Table S4**: The B_max_ and pK_d_ values obtained from saturation binding assays using [^3^H]-N/OFQ with HEK_NOP_ monoclonal cell lines, over several passages. Areas shaded in dark, indicate that the cell died between passages.

| Clone | GAPDH C_t_ | MOP C_t_ | Δ C_t_ Mean | GAPDH C_t_ | NOP C_t_ | Δ C_t_ Mean |
| --- | --- | --- | --- | --- | --- | --- |
| HEK_MOP/NOP_ #1 | 19.34 | 23.74 | 4.4 | 19.31 | 18.27 | -1.04 |
| HEK_MOP /NOP_ #2 | 24.27 | 26.71 | 2.44 | 25.05 | 21.98 | -3.07 |
| HEK_MOP /NOP_ #3 | 26.44 | 28.77 | 2.33 | 26.88 | 23.83 | -3.05 |
| HEK_MOP /NOP_ #5 | 27.61 | 30.09 | 2.48 | 28.06 | 34.95 | 6.9 |
| HEK_MOP /NOP_ #7 | 20.42 | 22.87 | 2.47 | 20.31 | 19.19 | -1.12 |

**Table S5**: C_t_ values for HEK_MOP /NOP_. Clones shaded in grey showed low ΔC_t_ values and were screened using radioligand saturation binding. Samples were screened from single cell RNA extractions (n=1).


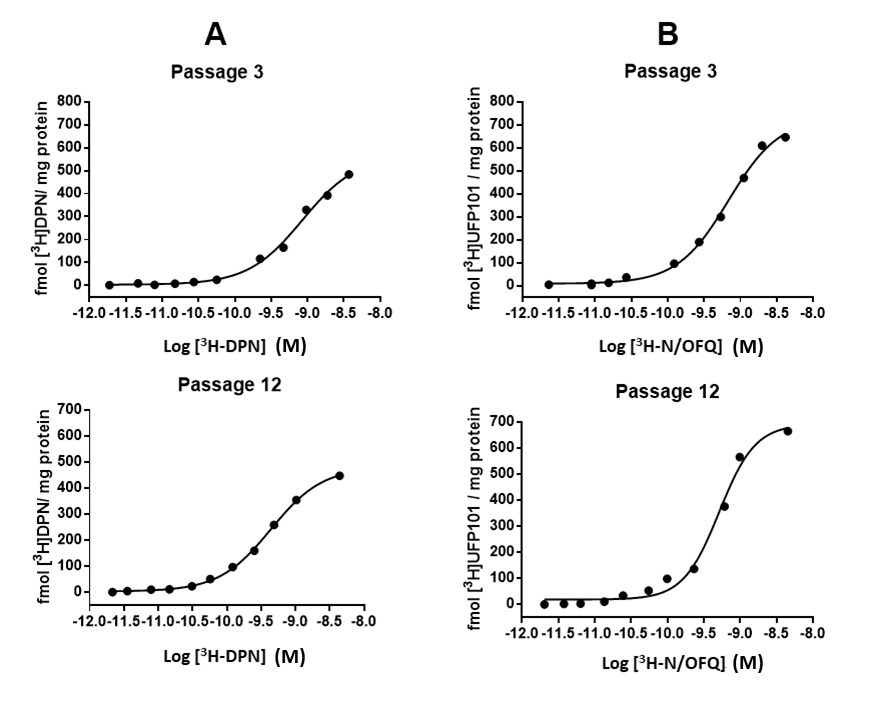


**Figure S3**: A) Representative saturation binding curves using [^3^H]-DPN for receptor expression in HEK_MOP /NOP_ #1 clones assessed at passage 3 and passage 12. B) Representative saturation binding using [^3^H]-N/OFQ curves depicting receptor expression in HEK_MOP /NOP_ #1 clones assessed at passage 3 and passage 12.

|  | Passage 3 | | Passage 12 | |
| --- | --- | --- | --- | --- |
|  | pK_d_ | B_max_ | pK_d_ | B_max_ |
| [^3^H]-DPN | 8.98 | 502.8 | 8.72 | 464 |
| [^3^H]-N/OFQ | 9.07 | 807.7 | 9.29 | 787.6 |

**Table S6:** The B_max_ and pK_d_ values obtained from saturation binding assays with HEK_MOP /NOP_ monoclonal cell lines, over several passages.

**2. De101 Structure and Chemistry.**

**Figure S4:** The chemical structure of the bivalent pharmacophore, De101.

Analytical HPLC analyses was performed on a Beckman 116 liquid chromatograph equipped with a Beckman 166 diode array detector. The purity of DermATTO488 was monitored at 220 nm and assessed with a Symmetry C18 column (4.6 x 75 mm, 3.5μm particle size) at a flow rate of 0.5 mL/min using a linear gradient from 100% of A (water + 0.1% trifluoacetic acid) to 100% of B (acetonitrile + 0.1% trifluoacetic acid) over a period of 25 minutes. The molecular weight of the conjugated peptide was confirmed by ESI Micromass ZQ, Waters.


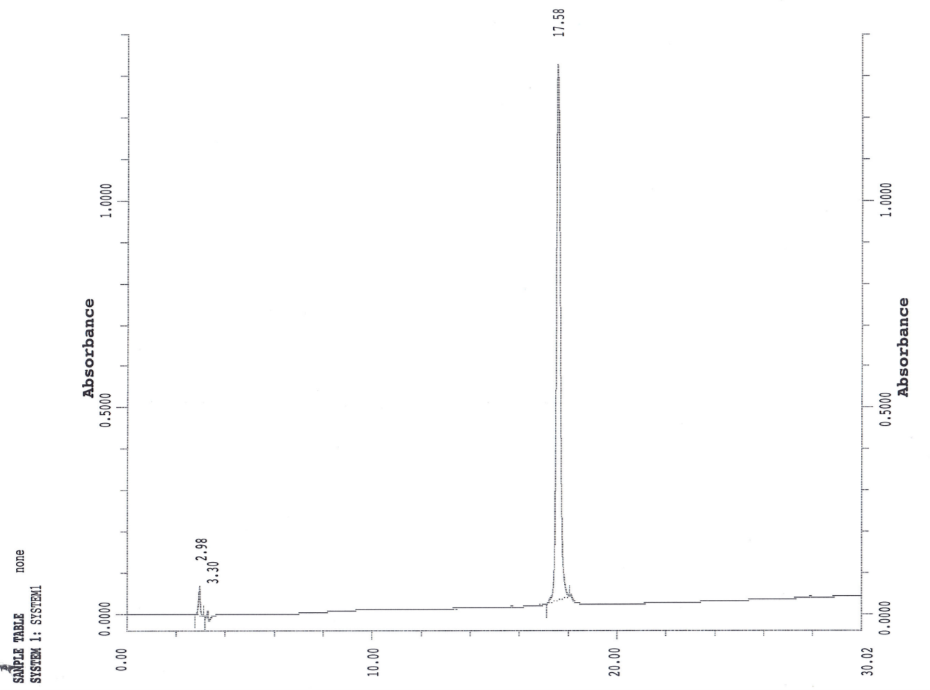


**Figure S5**: Analytical HPLC profile of De-101

**Figure S6**: Mass spectra of De-101, MW calculated 3107.56 Da; MW found 3107.67 Da

**3. Exploring peptide linkage**

As mentioned in the main text, experiments were undertaken to determine how activation of the MOP/NOP pairing reacted by co-incubation with monovalent ligands (**Figure S7**).


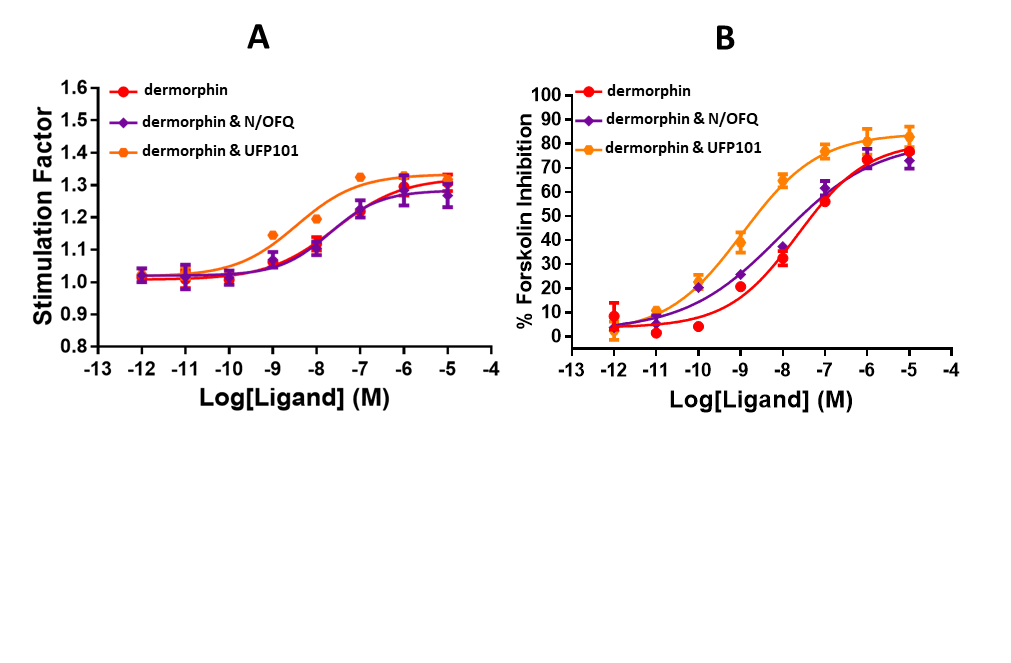
**Figure S7**: (A) Ligand stimulated GTPγ[^35^S] binding by dermorphin alone, dermorphin co-incubated with N/OFQ or with UFP-101 in HEK_MOP/NOP_ cell membranes. (B) Ligand stimulated inhibition of forskolin-induced cAMP production of dermorphin alone, dermorphin co-incubated with N/OFQ or with UFP-101 in HEK_MOP/NOP_ cells. Data are the mean (±SEM) for n=5 experiments.

**3. Determination of Opioid receptor mRNA expression in mouse hippocampal CA1 cells**

CA1 hippocampal tissue was obtained as mentioned in main paper methods. To determine CA1 opioid receptor expression qPCR experiments [2] were performed using the housekeeper (HK) gene GUSB and Taqman probes for mouse MOP, DOP, KOP and NOP mRNA. As shown in **Table S7** mRNA for all opioid receptors was expressed with highest levels for MOP and NOP. It is worthy of mention that mRNA relative abundance is no indication of receptor protein relative abundance.

|  | **HK-GUSB C_t_** | **Opioid C_t_** | **ΔC_t_** |
| --- | --- | --- | --- |
| **MOP** | 27.01±0.13 | 24.63±0.27 | -2.38±0.19 |
| **NOP** | 24.18±0.17 | 24.97±0.13 | 0.58±0.03 |
| **DOP** | 26.94±0.15 | 31.85±0.44 | 4.91±0.29 |
| **KOP** | 27.84±0.71 | 30.12±0.28 | 2.28±0.9 |

**Table S7**: qPCR analysis of CA1 tissue comparing HK gene GUSB to opioid receptor of interest. ΔC_t_ is defined as gene of interest Cycle threshold (C_t_)- Housekeeper gene C_t_. Data are the mean ± SEM of 4 experiments.

**Supplementary References**

1. Bird, M.F., et al., Characterisation of the Novel Mixed Mu-NOP Peptide Ligand Dermorphin-N/OFQ (DeNo). PLoS One, 2016. **11**(6): p. e0156897.

2. Al-Hashimi, M., et al., Evidence for nociceptin/orphanin FQ (NOP) but not micro (MOP), delta (DOP) or kappa (KOP) opioid receptor mRNA in whole human blood. Br J Anaesth, 2016. **116**(3): p. 423-9.
