# Supplementary material for: MOP and NOP receptor interaction: Studies with a dual expression system and bivalent peptide ligands
Source: PLoS One. 2022 Jan 21;17(1):e0260880. doi: 10.1371/journal.pone.0260880 (PMC8782398; doi:10.1371/journal.pone.0260880)
Supplement: S1 Raw images — (PDF) [file pone.0260880.s002.pdf]

Western blot Raw images

All images were obtained by using BioRad Imagelab software as detailed in main text methods section. Images are presented in the order they appear in Figure 11 of the main text. Molecular weights of the biotyinlated ladder (Cell signalling. Product#7727) are included in text. The red box indicates the area chosen to be used in Figure 11.

HEK_{MOP} Western blot results

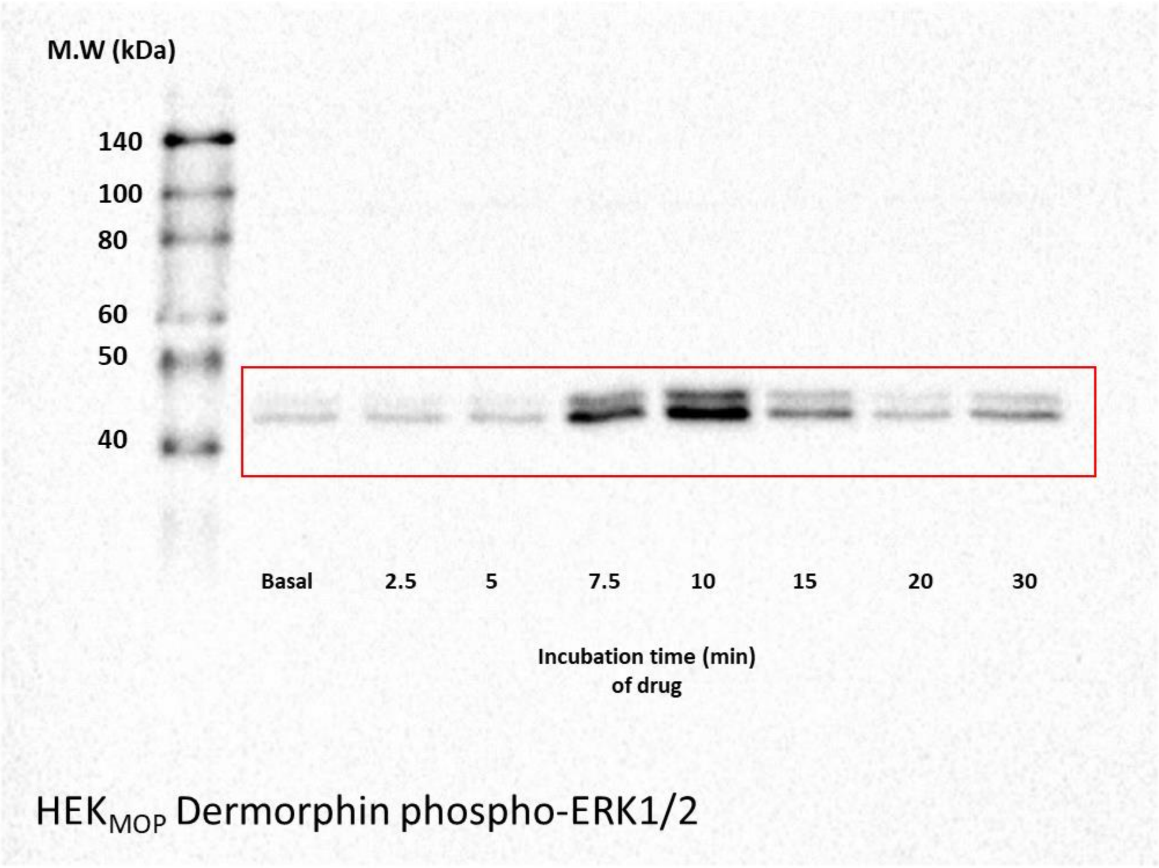

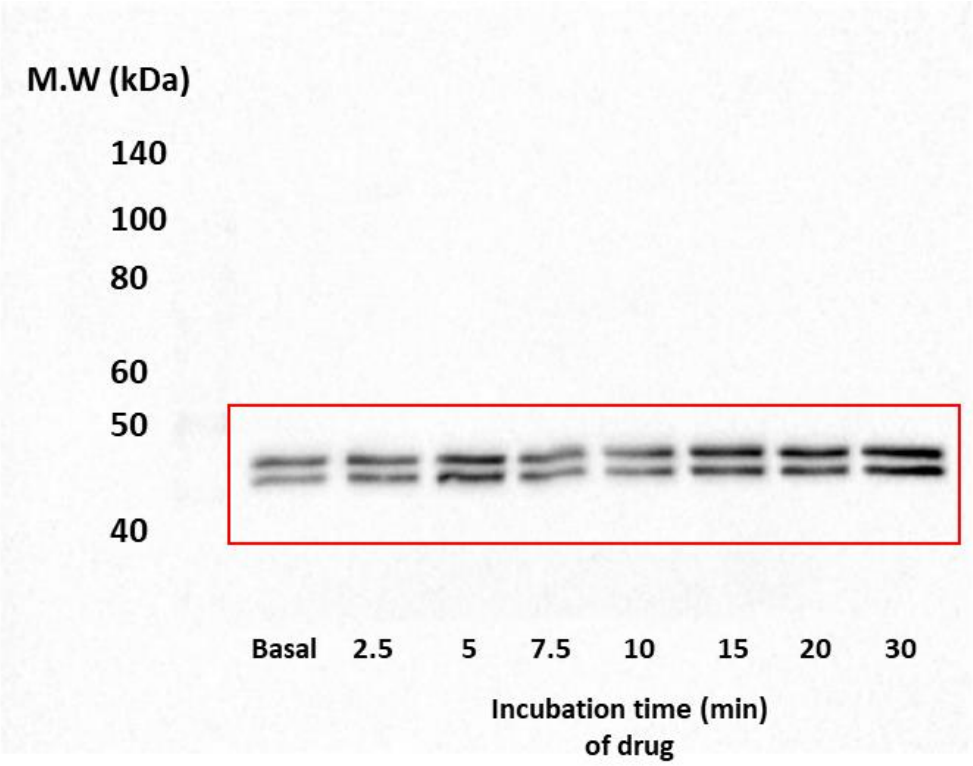

HEK$_{MOP}$ Dermorphin Total ERK1/2

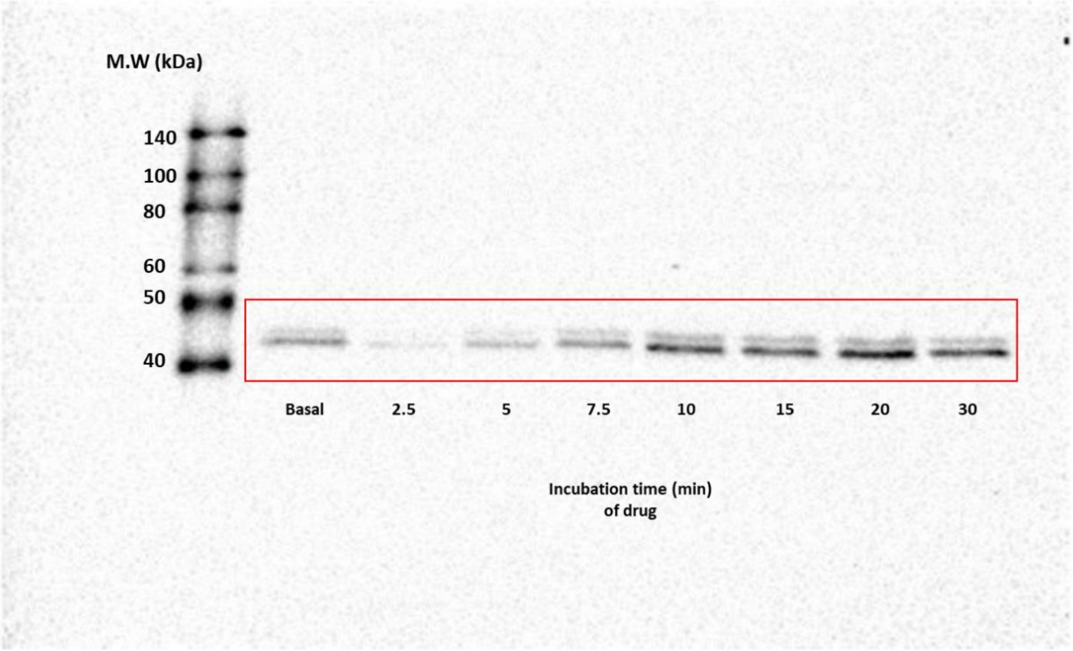

HEK$_{MOP}$ DeNO phospho-ERK1/2

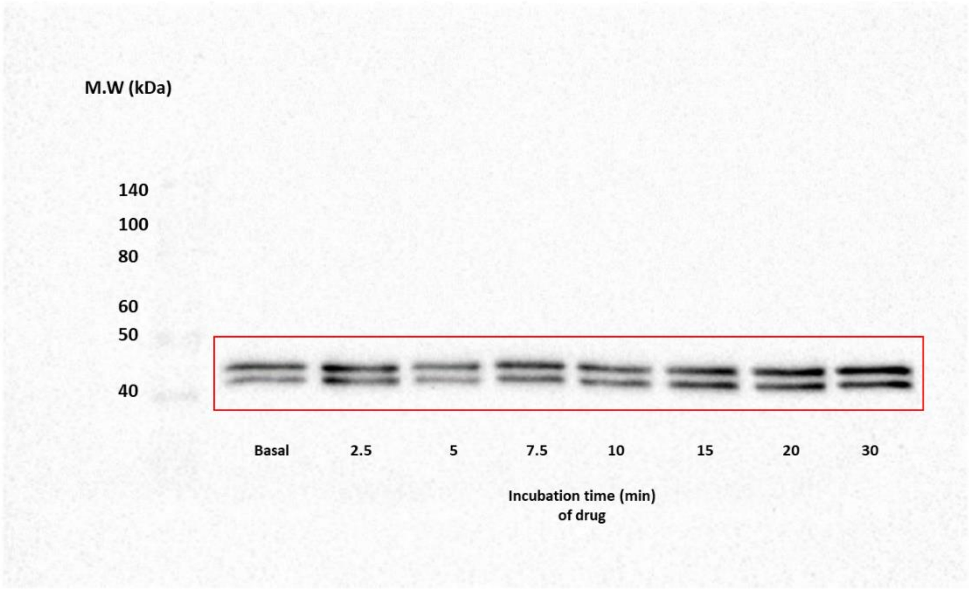

HEK$_{MOP}$ DeNO Total ERK1/2

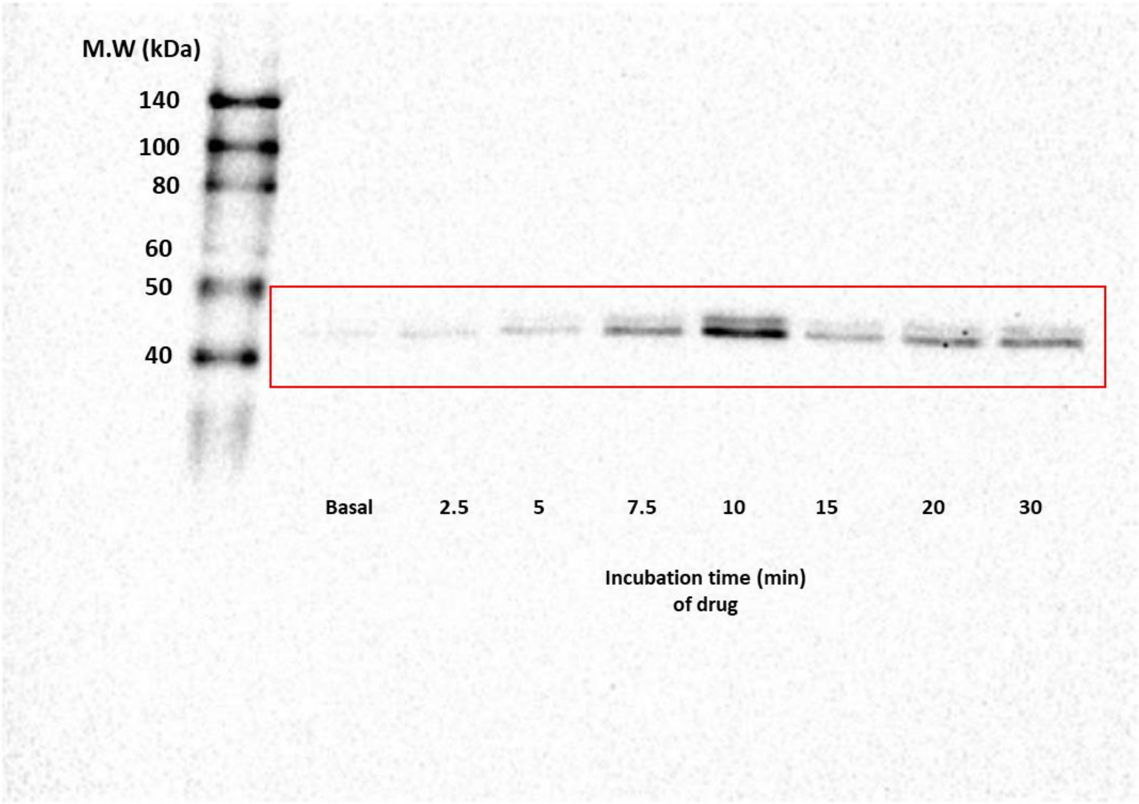

HEK$_{MOP}$ De101 phospho-ERK1/2

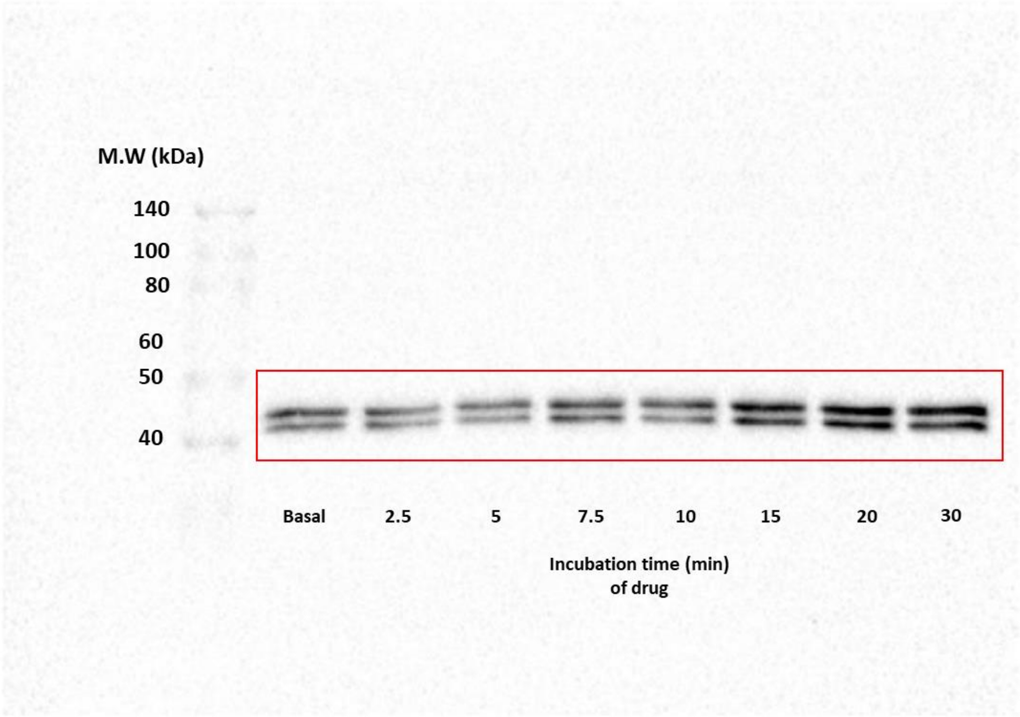

HEK$_{MOP}$ De101 Total ERK1/2

<u>HEK$_{NOP}$ Western blot results</u>

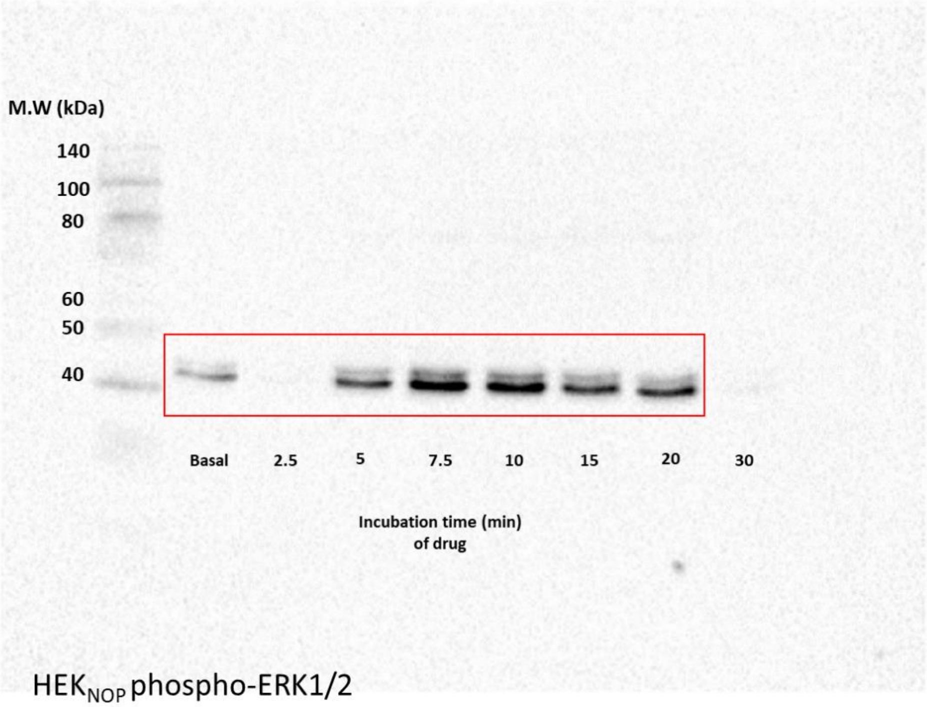

HEK$_{NOP}$ phospho-ERK1/2

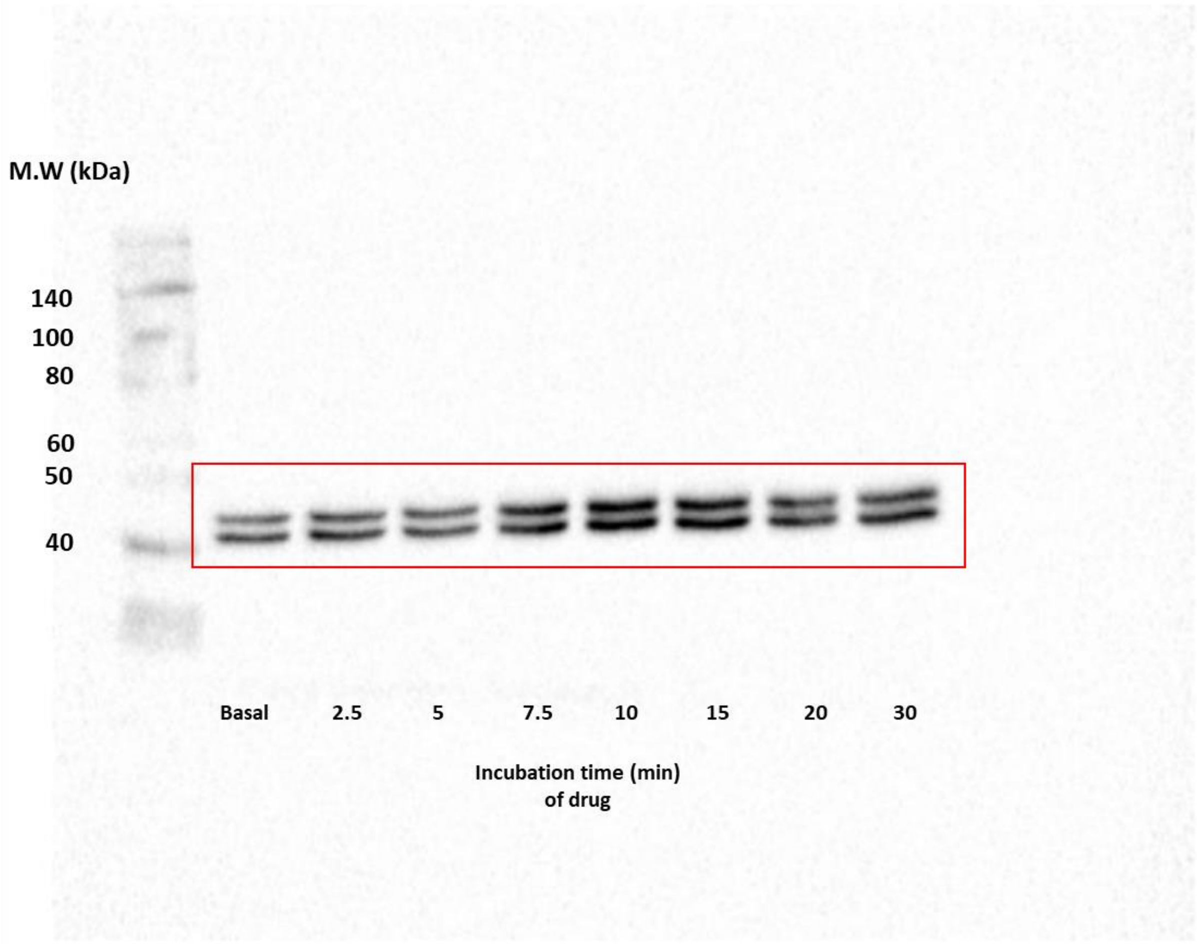

HEK$_{NOP}$ N/OFQTotal ERK1/2

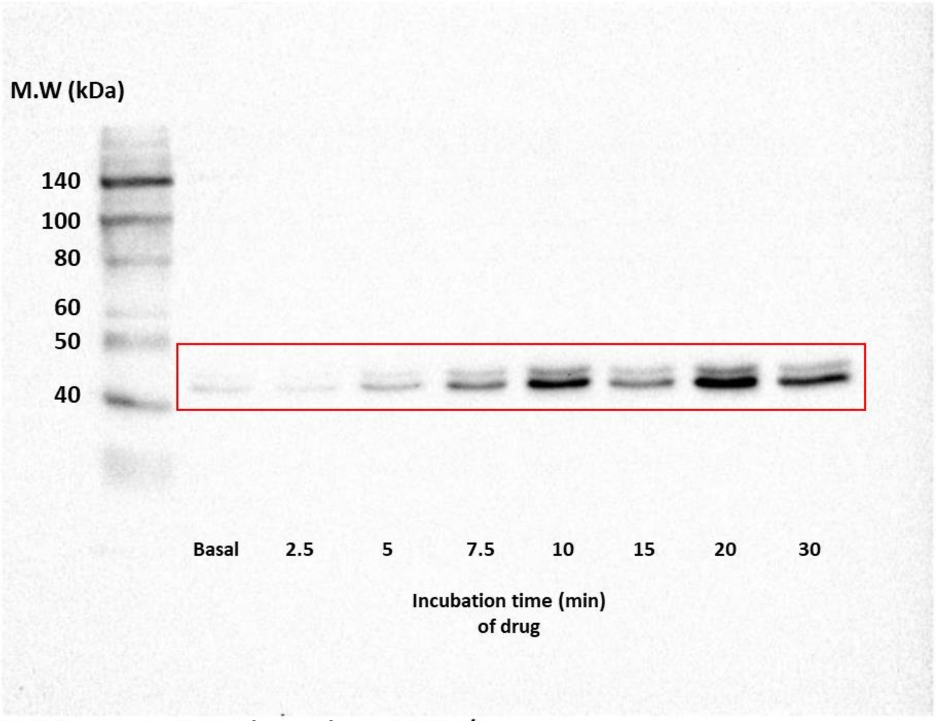

HEK$_{NOP}$ DeNO phospho-ERK1/2

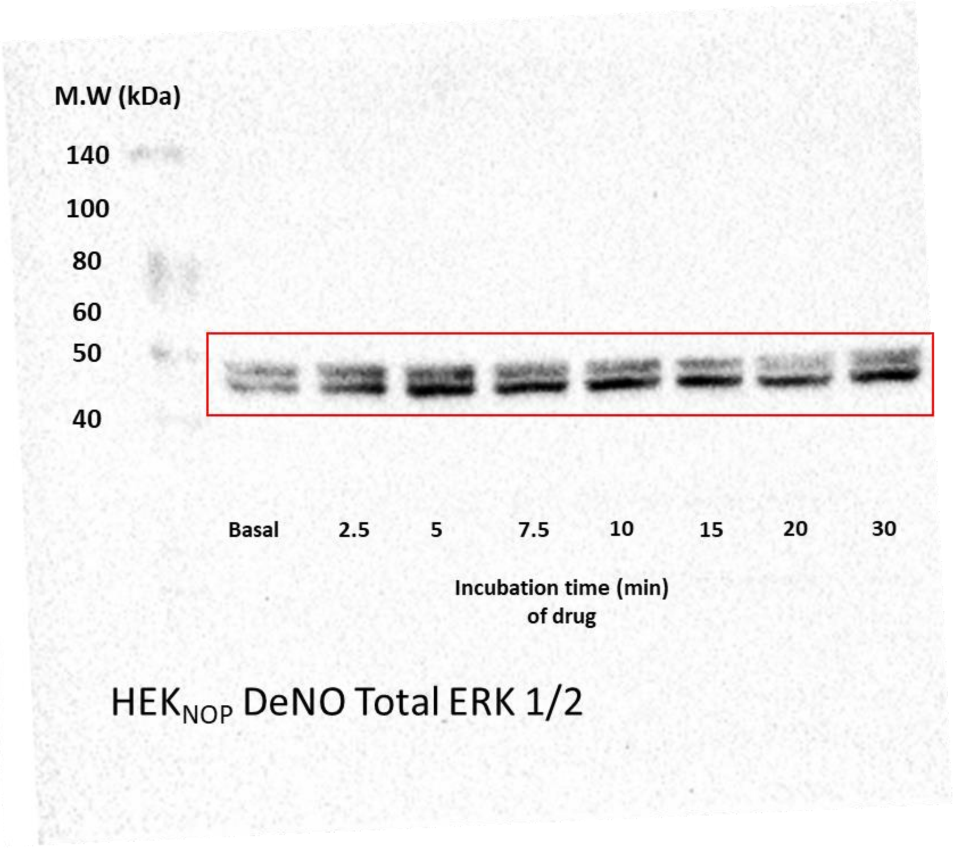

HEK$_{NOP}$ DeNO Total ERK 1/2

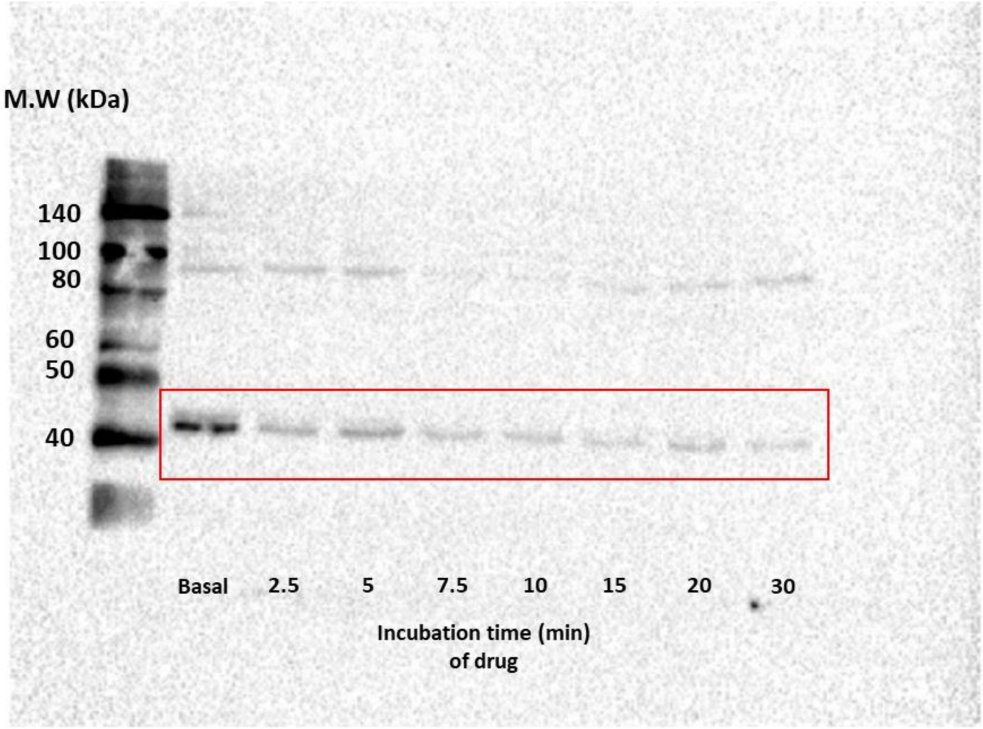

HEK$_{NOP}$ De101 phospho-ERK1/2

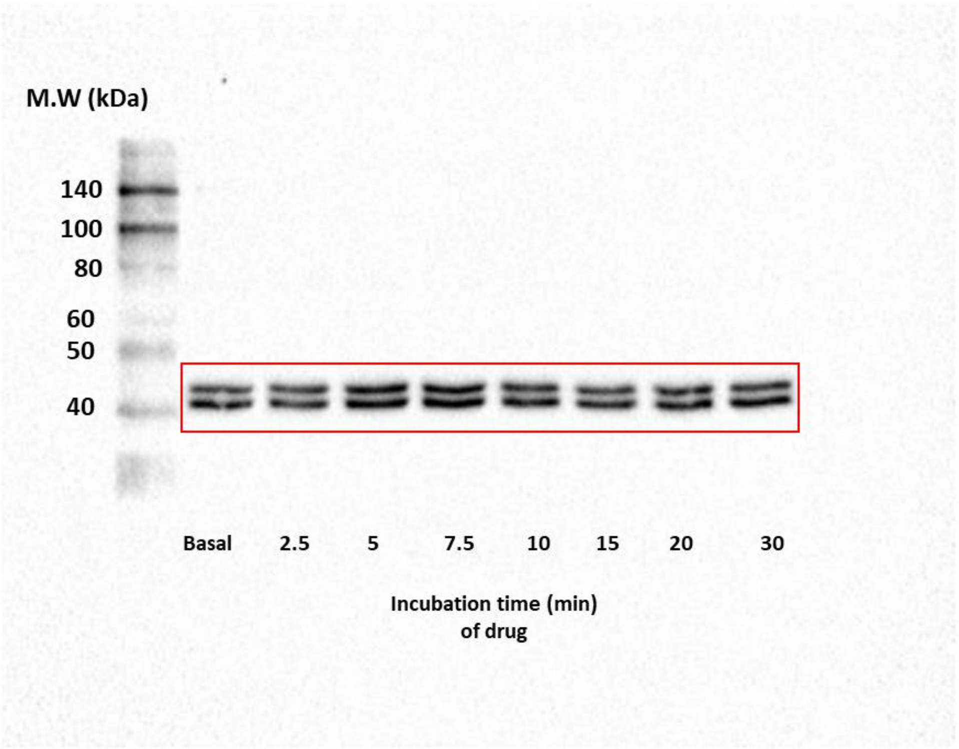

HEK$_{NOP}$ De101 Total ERK1/2

HEK$_{MOP/NOP}$ Western blot results

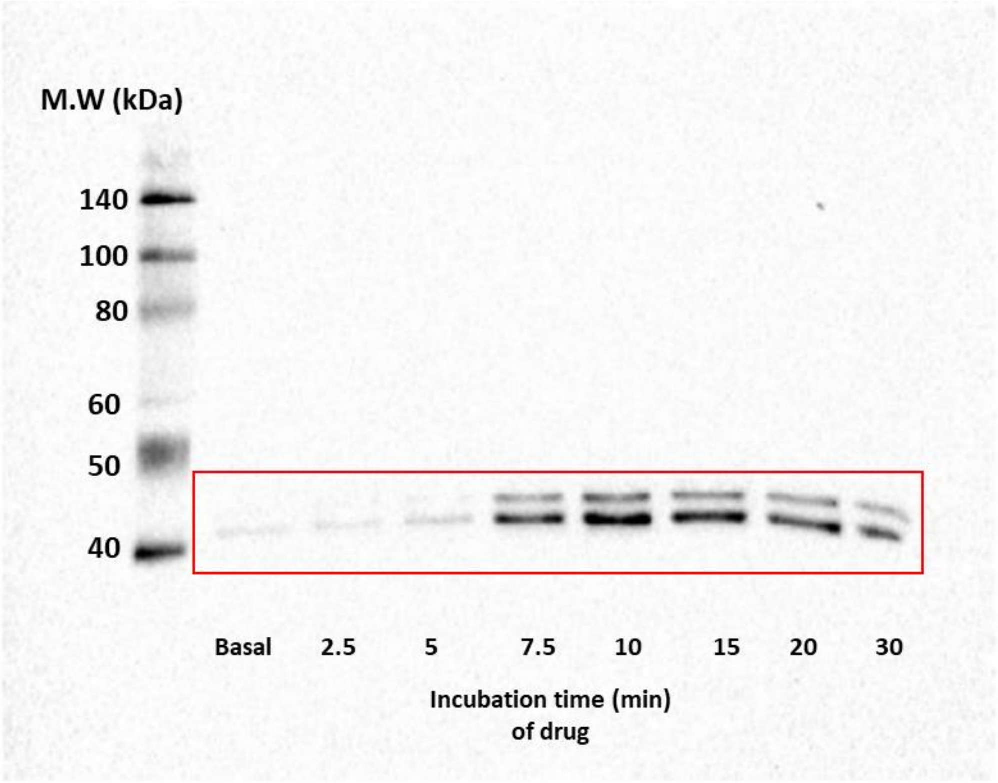

HEK$_{MOP/NOP}$ Dermorphin phospho-ERK1/2

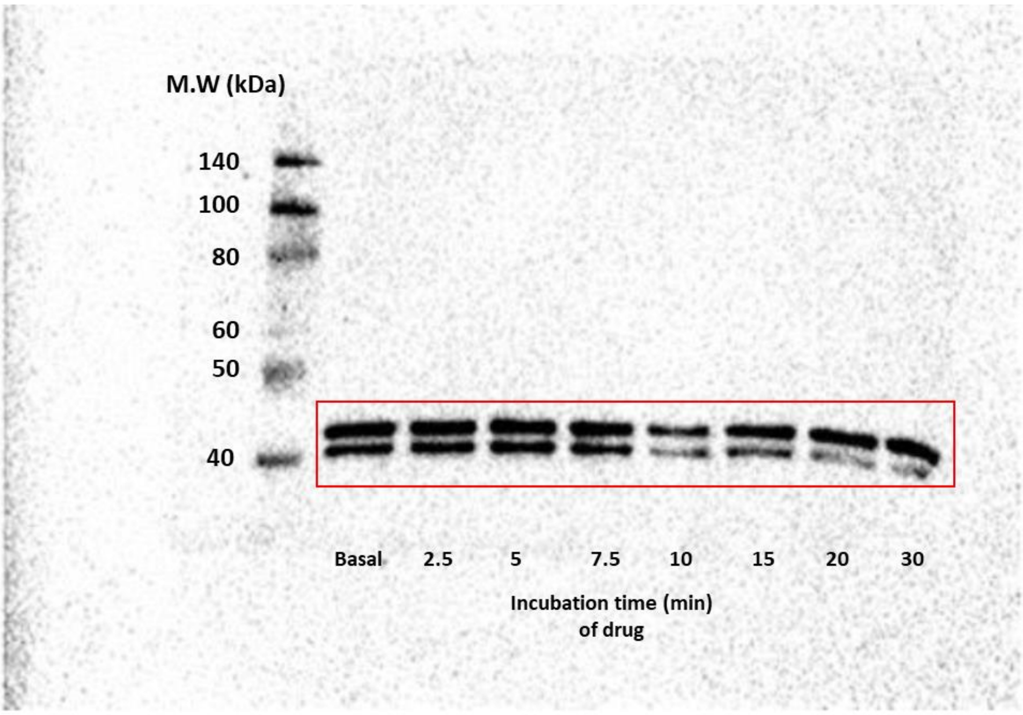

HEK$_{MOP/NOP}$ Dermorphin Total ERK1/2

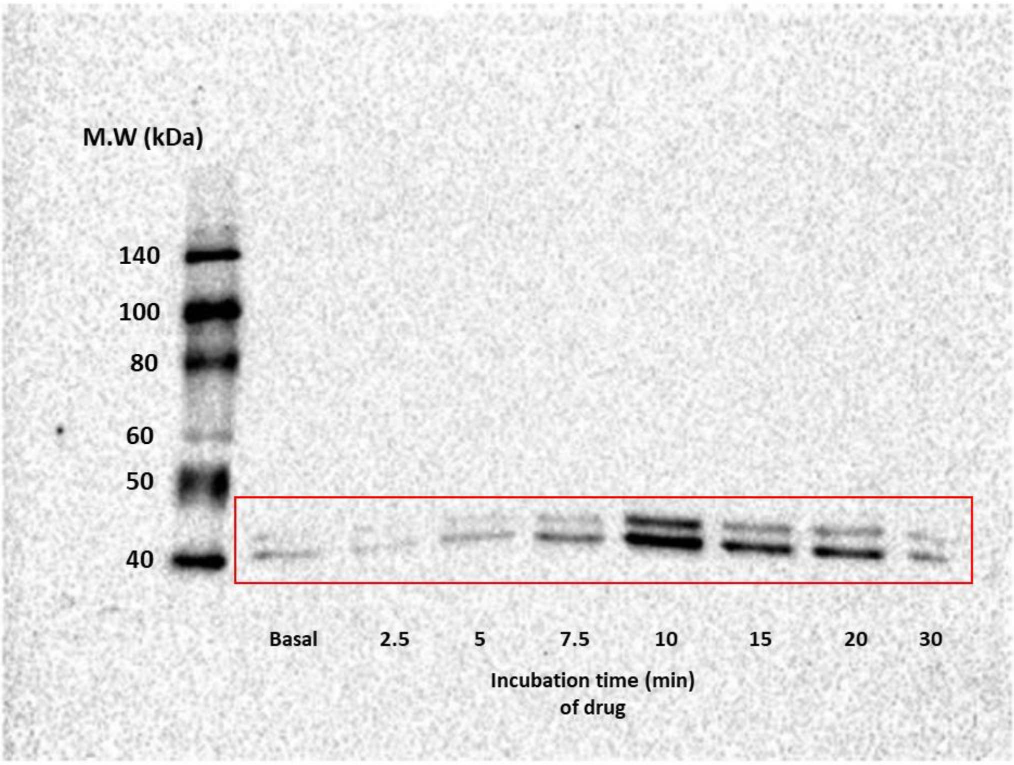

HEK$_{MOP/NOP}$ N/OFQ phospho-ERK1/2

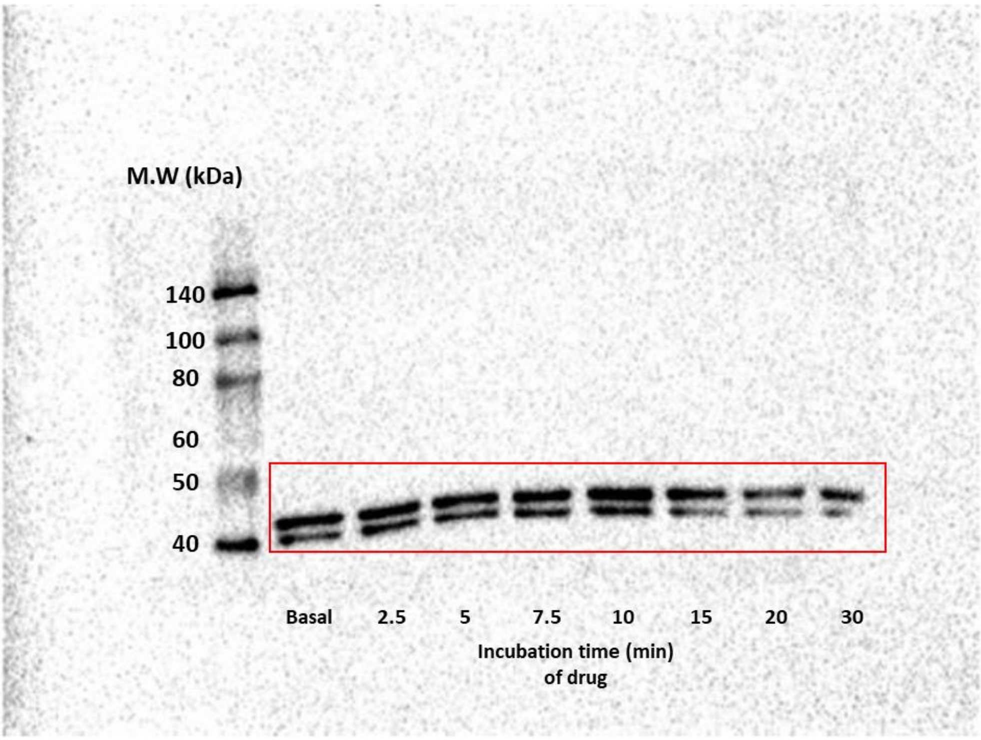

HEK$_{MOP/NOP}$ N/OFQ Total ERK 1/2

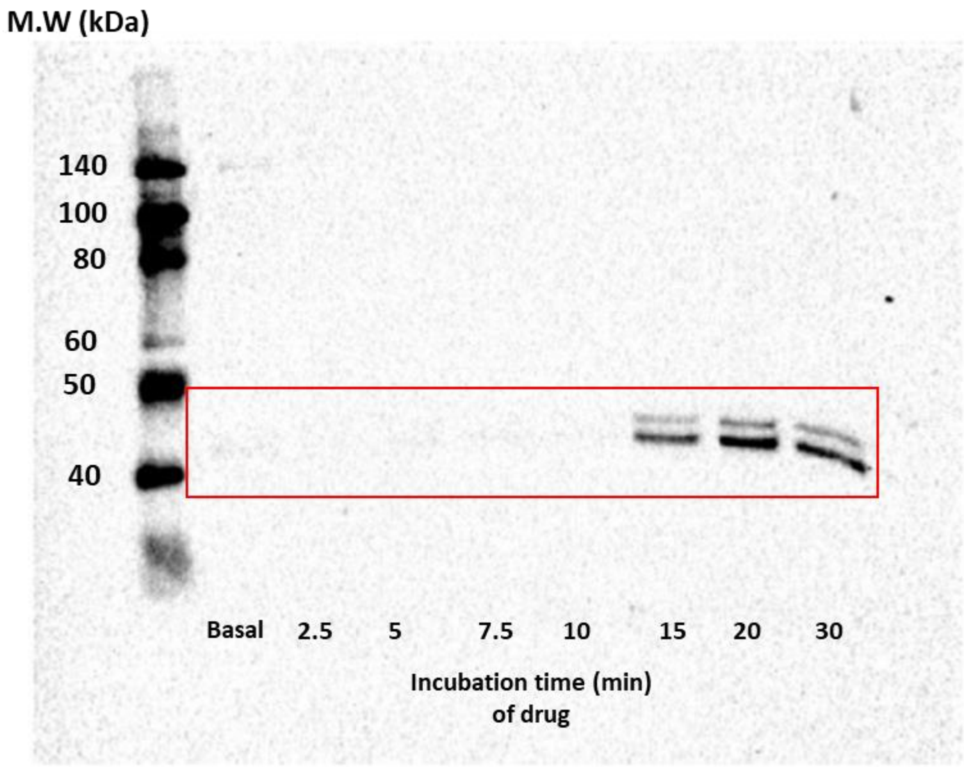

HEK$_{MOP/NOP}$ DeNO phosphor-ERK1/2

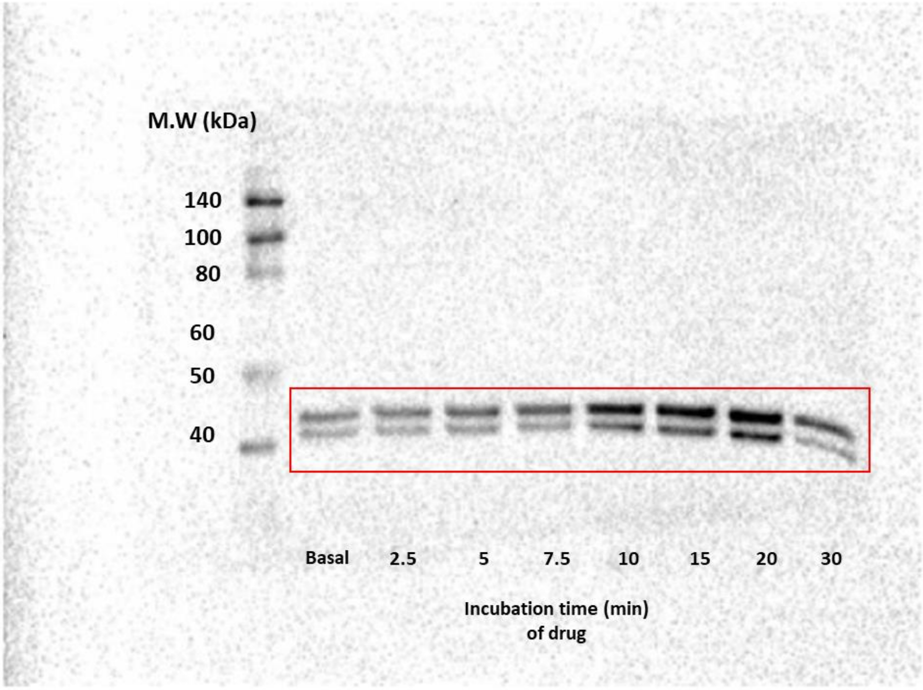

HEK$_{MOP/NOP}$ DeNO Total ERK1/2

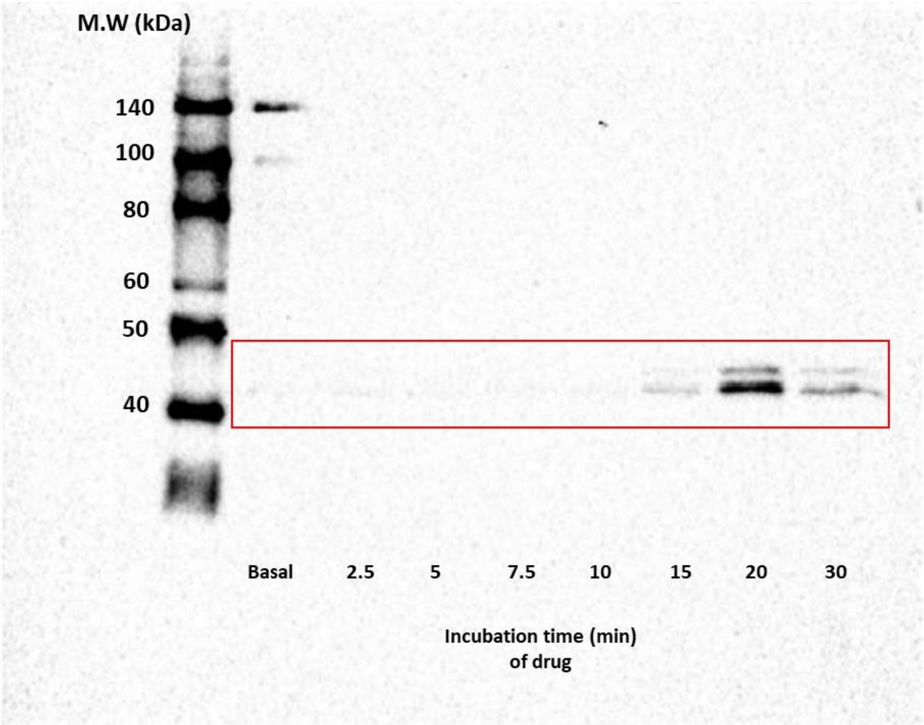

HEK$_{MOP/NOP}$ De101 phospho-ERK1/2

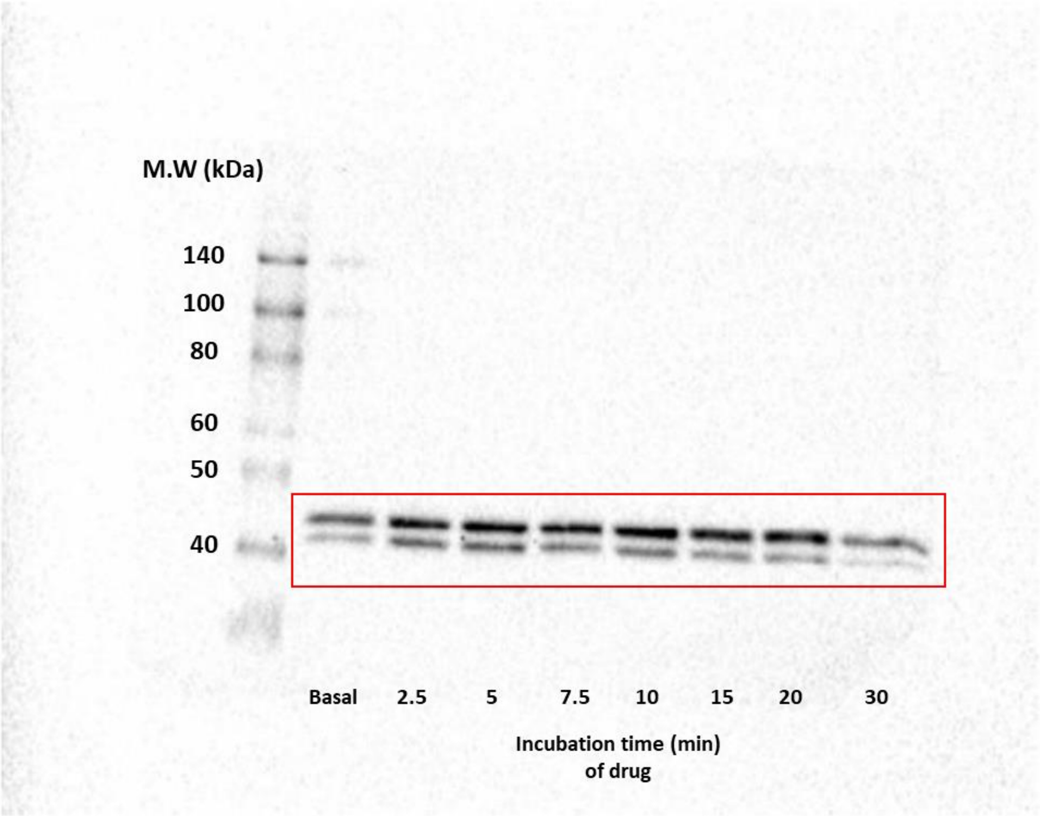

HEK$_{MOP/NOP}$ De101 Total ERK1/2
